# Effects of respiratory virus vaccination and bovine respiratory disease on the respiratory microbiome of feedlot cattle

**DOI:** 10.3389/fmicb.2023.1203498

**Published:** 2023-06-13

**Authors:** Taylor B. McAtee, Lee J. Pinnell, Sherri A. Powledge, Cory A. Wolfe, Paul S. Morley, John T. Richeson

**Affiliations:** ^1^Department of Agricultural Sciences, West Texas A&M University, Canyon, TX, United States; ^2^VERO Program, Texas A&M University, Canyon, TX, United States

**Keywords:** bovine respiratory disease, respiratory microbiome, respiratory vaccination, beef cattle, intranasal vaccination, randomized clinical trial

## Abstract

**Introduction:**

The objectives of this study were to evaluate the impacts of two modified-live virus (MLV) vaccination protocols and respiratory disease (BRD) occurrence on the microbial community composition of the nasopharynx in feedlot cattle.

**Methods:**

The treatment groups included in this randomized controlled trial included: 1) no viral respiratory vaccination (CON), 2) intranasal, trivalent, MLV respiratory vaccine in addition to a parenteral BVDV type I and II vaccine (INT), and 3) parenteral, pentavalent, MLV respiratory vaccination against the same agents (INJ). Calves (*n* = 525) arrived in 5 truckload blocks and were stratified by body weight, sex, and presence of a pre-existing identification ear-tag. A total of 600 nasal swab samples were selected for DNA extraction and subsequent 16S rRNA gene sequencing to characterize the microbiome of the upper respiratory tract. Nasal swabs collected on d 28 from healthy cattle were used to evaluate the impact of vaccination on upper respiratory tract (URT) microbial communities.

**Results:**

Firmicutes were less abundant in INT calves (*n* = 114; *P* < 0.05) and this difference was attributed to decreased relative abundance (RA) of *Mycoplasma spp*. (*P* = 0.04). *Mannheimia* and *Pasteurella* had lower RA in INT (*P* < 0.05). The microbiome in healthy animals on d 28 had increased Proteobacteria (largely *Moraxella* spp.) and decreased Firmicutes (comprised almost exclusively of *Mycoplasma spp.*) compared to animals that were treated for or died from BRD (*P* < 0.05). Cattle that died had a greater RA of *Mycoplasma spp*. in their respiratory microbiome on d 0 (*P* < 0.02). Richness was similar on d 0 and 28, but diversity increased for all animals on d 28 (*P*>0.05).

## 1. Introduction

Bovine respiratory disease (BRD) is the most common reason for antimicrobial drug treatment among feedlot cattle in North America (Brault et al., 2019; White and Larson, 2020), and BRD reportedly impacts an estimated 16.2% of feedlot cattle (USDA Feedlot, 2011). The pathogenesis of BRD is considered a classic example of a multifactorial disease that is caused by a combination of different viral and bacterial pathogens, along with host and environmental factors. Cattle experience many different stressors through the marketing system including environmental, dietary, handling, transportation, weaning, and commingling. The bacterial agents, namely *Mannheimia haemolytica, Pasteurella multocida*, and *Histophilus somni* (*Hs*), are widely considered to be the most important causes of acute onset pneumonia (Plummer et al., 2004). However, most studies investigating the microbial agents of BRD have been culture-based and frequently target only these pathogens (Duff and Galyean, 2007; Fulton and Confer, 2012; Mosier, 2014; Lubbers and Turnidge, 2015). Other investigations have identified a much broader set of commensal bacteria, but this has not altered the perception of the most important agents causing BRD (Booker et al., 2008; Klima et al., 2019; Valeris-Chacin et al., 2022).

Respiratory disease in cattle is thought to be associated with changes in the respiratory microbiome (Chai et al., 2022; Crosby et al., 2022), but our understanding of these shifts and their potential implications is lacking. The microbial communities of the bovine respiratory tract are reportedly influenced by age (Timsit et al., 2017), antimicrobial use (Timsit et al., 2017), diet (Hall et al., 2017), and environment (Timsit et al., 2017), and it is speculated that host genetics, management strategies, season, and vaccinations are also influential (Zeineldin et al., 2019). The composition of respiratory microbial communities in cattle is also altered by the physiological stress associated with transport and commingling (Timsit et al., 2020). Recent research suggests that disturbances to healthy upper respiratory tract (URT) microbial communities create ecological niches for opportunistic bacterial pathogens to occupy (Kaul et al., 2020). The respiratory microbiome experiences a composition shift after transport to a feedlot, and microbial communities in the URT are most variable during the first 60 days (Amat et al., 2019). This timeframe overlaps when cattle are most susceptible to BRD (Booker et al., 2008) and further emphasizes the need to characterize whole microbial communities when investigating respiratory disease in cattle.

Nasopharyngeal microbial communities have been previously characterized using various diagnostic modalities, but the impact of respiratory vaccines administered *via* different routes (i.e., intranasal or parenteral) is unknown. In the feedlot setting, vaccines administered against viral respiratory agents contain modified-live virus (MLV) antigens that can be administered either intranasally or parenterally, with different immunological responses expected. Parenteral vaccination primarily induces a systemic immune response within the host (Rayevskaya and Frankel, 2001), while intranasal vaccination is designed to specifically stimulate immune responses in the nasal epithelium with the primary goal of antigen-specific secretory IgA production (Holmgren and Czerkinsky, 2005). Research in humans suggests that viral regulation of microbial community composition in the URT may facilitate the transition of important members from pathobiont to a pathogen (Hanada et al., 2018) and has shown that the enrichment of viral agents in the URT resulted in specific microbial taxa impacting the host immune response (Sonawane et al., 2019). Additionally, investigators have previously described the potential synergism between *Hs* and bovine respiratory syncytial virus (BRSV) in affecting BRD occurrence (Gershwin et al., 2005; Corbeil, 2007). However, published investigations of the impacts of MLV vaccination on URT microbial communities are unavailable. The immunomodulatory effects of BRSV may contribute to dysbiosis through emphasis on shifting a Th2 immunological response and increasing *Hs* in the respiratory tract of cattle (Powledge et al., 2022).

The primary objective of this study was to compare the bacterial community composition in the nasopharynx of feedlot cattle that were administered MLV respiratory vaccines as part of a randomized controlled trial by intranasal or parenteral route and in unvaccinated cattle. The secondary objectives were to evaluate differences in the URT microbiome among feedlot cattle with different health statuses over time.

## 2. Materials and methods

### 2.1. Study overview

A randomized, controlled trial was used to compare the impacts of two respiratory virus vaccination protocols on cattle not vaccinated against respiratory viruses (CON). One vaccination group received an intranasal MLV respiratory virus vaccine containing bovine herpes virus type 1 (BHV1), BRSV, and parainfluenza virus type 3 (PIV3) and a parenteral vaccine containing bovine viral diarrhea virus (BVDV) types I and II (INT), and a group vaccinated parenterally against the same agents using a pentavalent MLV respiratory vaccine (BHV1, BRSV, PIV3, and BVDV types I and II—INJ). Samples of respiratory secretions in the nasopharyngeal region were obtained on d 0 and d 28 and upon initial treatment for BRD. Samples were analyzed using 16S rRNA gene sequencing to characterize microbial community structures and determine differences among vaccine treatment groups, among cattle with different health outcomes, and over time. Administration and handling of vaccines followed label and Beef Quality Assurance guidelines. Animal use protocols were approved before the initiation of the study by the West Texas A&M University (WTAMU) Institutional Animal Care and Use Committee (IACUC #2020.10.002).

### 2.2. Treatment groups

A total of 525 crossbred beef bull and steer calves (213 ± 18.4 kg) were acquired from auction markets in central Texas and enrolled in the study. Cattle were randomly allocated to experimental treatment pens (*n* =12 per pen, 15 pens per treatment) (CON *n* = 175; INJ *n* = 175; INT *n* = 175), stratified by truckload (*n* = 5), body weight, sex (bull [*n* = 129] or steer [*n* = 396]), and the presence of a pre-existing identification ear tag. As such, these factors were homogenously represented across pens within truckload blocks and cattle housed in a pen belonged to the same vaccine treatment group. The INT vaccination group received a trivalent MLV respiratory vaccine administered intranasally (Inforce 3, Zoetis) and a parenteral vaccine containing BVDV types I and II given subcutaneously (Bovi-shield BVD, Zoetis). The INJ group received a parenteral, pentavalent MLV respiratory vaccine given subcutaneously (Bovi-shield GOLD 5, Zoetis). To minimize the potential transmission of infectious agents between treatment groups (including vaccine-origin viruses), an empty pen was maintained between treatment groups to eliminate direct contact. Additionally, initial processing, subsequent sample collection, and BRD treatments were conducted in the order of CON, INJ, and then INT treatment groups to minimize the potential for vaccine or natural virus transmission.

### 2.3. Study population and cattle management

This study was conducted from November 2020 to May 2021 at the WTAMU Research Feedlot, located near Canyon, TX. Upon feedlot arrival (d−1), individual body weight (BW), sex (bull or steer), and the presence of a pre-existing ear tag were recorded (Munoz et al., 2020). Each animal was affixed with unique visual and radio frequency ear tags. All cattle were administered a growth-promoting implant containing 200 mg progesterone, 20 mg estradiol benzoate, and 29 mg tylosin tartrate (Component E-S with Tylan, Elanco Animal Health), an injectable clostridial bacterin-toxoid vaccine (Calvary 9, Merck Animal Health), and injectable, and oral anthelmintics (Ivermax Plus, Aspen, and Valbazen, Zoetis, respectively). The following day (d 0), in addition to the MLV vaccine treatments described above, cattle were administered a *Mannheimia haemolytica* bacterin-toxoid (One Shot, Zoetis) and tildipirosin as a metaphylactic antimicrobial drug (Zuprevo, Merck Animal Health). Bulls were band castrated (Callicrate, No-Bull Enterprises) and treated with an analgesic (meloxicam, 1 mg/kg BW P.O., Unichem Pharmaceuticals).

Trained personnel that were blinded to treatment group assignments monitored the health of cattle each morning and assigned a clinical illness score (CIS) to each animal. A CIS of 1 described a “normal” steer without any visible clinical signs of sickness. Steers with a CIS of 2 represented a “moderately ill” steer, which appeared gaunt, had nasal/ocular discharge, lagged behind others, and coughed. A CIS score of 3 indicated a “severely ill” steer with purulent nasal/ocular discharge, labored breathing, and severe depression. Lastly, CIS 4 corresponded to a “moribund” steer that was unresponsive to human approach and near death. Animals assigned as CIS of 2 or 3 were removed from the pen and further examined, including measurement of rectal temperatures. Steers assigned CIS of 3 or 4 were classified as BRD cases, as were animals assigned CIS of 2 if they also had a rectal temperature ≥39.7°C. Cattle classified as CIS 4 were euthanized. Because tildipirosin was administered metaphylactically on d 0, no other antimicrobial drug (AMD) treatments were administered until after d 7. After this period, BRD cases were treated with an AMD, using a standardized treatment protocol (all AMDs were stored and administered according to label guidelines). Steers first diagnosed with BRD were treated with florfenicol (Nuflor, Merck Animal Health). Following the expiration of a 3-day post-treatment interval (PTI), steers again meeting the same BRD case definition were treated with enrofloxacin (Baytril, Elanco Animal Health). After another 3-day PTI, steers meeting the BRD case definition a third time were eligible for a third and final treatment with ceftiofur crystalline-free acid (Excede, Zoetis). Steers that received three antimicrobial treatments with < 0.45 kg ADG since d 0 or body condition score < 3 of 9 were removed from the study and considered chronically ill. Cattle that died or were euthanized underwent post-mortem examination, and the cause of death was recorded. Further information about the study population, cattle handling, and the health and performance of cattle is described elsewhere (Powledge et al., 2022). The overall cumulative incidences for BRD, classification as chronically ill, and mortality were not statistically different among treatment groups (first BRD treatment, CON = 54.6%, INJ = 58.4%, INT = 58.9%, *P* = 0.83; chronic illness, CON = 7.4%, INJ = 5.1%, INT = 2.9%, *P* = 0.17; mortality, CON = 10.9%, INJ = 7.6%, INT = 5.2%; *P* = 0.37; Powledge et al., 2022). Cumulative health status during the 72-day study period was categorized to facilitate analysis of associations with URT microbiome composition. Animals that died from BRD-related causes were classified as “mortalities”; other cattle that were treated with AMD at any time for BRD were classified as “BRD cases,” and the remaining untreated cattle were classified as “healthy”.

### 2.4. Nasopharyngeal swab collection

On d 0 and d 28 for all calves, and at first treatment for BRD in affected calves, the nasal passage and nasopharynx were sampled using a 40 cm, rayon-tipped proctology swab (DNS, Puritan; Crosby et al., 2022). Before collection, the nasal planum and external nares were wiped with a clean paper towel to remove debris. Wearing a new exam glove, swabs were passed through the nostril to the nasopharynx and rotated at least three times against the mucosal surfaces. The swab tip was then placed in a sterile tube containing 1.5 ml of 100% ethanol. Whole-blood samples were also collected on days 0, 14, 28, 42, 56, and 70 to provide serum for analysis of BRSV antibody titer. Samples were immediately placed on ice, transported to the Texas A&M University Veterinary Education, Research, and Outreach laboratory (VERO; Canyon, TX), and stored at −80°C until further processing. From the total of 1,184 swabs samples collected, a subset (*n* = 600) was selected for DNA isolation and characterization by 16S rRNA gene sequencing (Table 1). For subset selection, three animals that were never treated for BRD and had the greatest average daily weight gain within their pen were selected from each pen. In addition, three steers classified as BRD cases were selected from each pen, stratifying on the number of BRD treatments that were administered to ensure equivalent distribution across pens, and thus vaccine treatment groups. Finally, DNS collected on d 0 and d 28 from cattle classified as chronically ill and those that died from bronchopneumonia were also used to characterize the respiratory microbiome. The *a priori* goal for sample size was to include 600 samples with 50 animals within each vaccine treatment group and within each health category (healthy, BRD cases, chronically ill, and mortalities). The available number of samples from chronically ill and mortalities did not total 50 for all vaccine treatment groups, and the remaining allotment for those samples was reassigned to the healthy and BRD case categories (Table 1).

**Table 1:** Numbers of nasopharyngeal samples tested, by treatment group, health status, and sampling timepoint (Total *n* = 561).

	**Treatment**			**Health status**		
**Subset**	**CON**	**INJ**	**INT**	**Healthy**	**BRD cases**	**Mortalities**
All cattle and timepoints	176	186	199			
Healthy cattle	70	77	84			
All D 0 samples	76	82	85	116	85	42
All D 28 samples	62	65	77	114	82	8
Healthy D 0 samples	35	41	41			
Healthy D 28 samples	35	36	43			

### 2.5. DNA isolation, 16S rRNA gene library preparation, and sequencing

A commercially available extraction kit (QIAamp PowerFecal DNA Kit, Qiagen) was used to isolate genomic DNA from DNS samples according to manufacturer instructions. Following isolation, DNA was quantified (ng·μL^−1^) using a Qubit 4 fluorometer (Thermo Fisher Scientific). After extraction, DNA was stored at −80°C until amplification was performed.

The V3-V4 region of the 16S rRNA gene was amplified using the 341f/785r primer pair (Klindworth et al., 2013). Amplification conditions were 98°C for 3 min, followed by 20 cycles of 98°C for 30 seconds, 55°C for 30 seconds, and 72°C for 1 min. Final elongation occurred at 72°C for 5 min. Amplicons were then purified using beads (AMPure XP beads, Beckman-Coulter), and sequencing libraries were prepared using the Nextera IDT kit (Illumina). Libraries were purified using AMPure XP beads and pooled in equal proportions based on molarities. The resulting pooled amplicon library was sequenced on an Illumina MiSeq instrument using 2 × 250 base pair (bp) paired-end chemistry at the Texas A&M Institute for Genome Sciences and Society sequencing core. Each plate of PCR reactions included a negative control, which consisted of an equal volume of nuclease-free sterile water as a template. These controls were included in the preparation of sequencing libraries. Sequencing of these negative control samples did not yield product, and therefore, they were not included in further downstream analysis.

### 2.6. BRSV-specific antibody titer analysis

Serum samples were collected from a randomly selected subset of 6 animals from each pen, as previously described (Powledge et al., 2022). In brief, relative to this investigation, whole blood collected by jugular venipuncture on d 0 and d 28 was centrifuged, and serum was frozen at −20°C until the determination of BRSV-specific serum neutralizing antibody titer as described by Rosenbaum et al. (1970).

### 2.7. Bioinformatics and descriptive analyses

Demultiplexed 16S rRNA gene sequence reads were imported into QIIME2 version 2022.2 (Bolyen et al., 2019). Amplicon sequence variants (ASVs) were generated using DADA2 (Callahan et al., 2016) which was used to filter reads for quality, remove chimeric sequences, and merge overlapping paired-end reads. Both forward and reverse reads were trimmed at 23 bp, while forward and reverse reads were truncated at 250 and 246 bp, respectively. Taxonomy was assigned using a Naive Bayes classifier trained on the SILVA 138 SSU NR 99 database (Quast et al., 2013), where sequences had been trimmed to include only the base pairs from the V3–V4 region bound by the 341f/785r primer pair. Reads mapping to chloroplast and mitochondrial sequences were filtered from the representative sequences and ASV table, and a midpoint-rooted phylogenetic tree was generated using default settings, which was used to calculate phylogeny-based metrics. Across all samples, 93.7% of ASVs were classified at the rank of genus, while over 99% of all ASVs were classified at each of the higher taxonomic ranks (i.e., family, order, class, and phylum).

Data and metadata were then imported into phyloseq (McMurdie and Holmes, 2013) using the “import_biom” function. Samples with less than 10,000 ASVs were omitted from the downstream analysis, which resulted in 559 samples with a range of 13,480 to 323,612 ASVs and an average of 71,798 ASVs per sample. Richness (observed ASVs) and Faith's phylogenetic diversity (FPD) were calculated for all samples with phyloseq and the “estimate_pd” function from the btools package in R. ASV counts for each sample were then normalized using cumulative sum scaling (Paulson et al., 2013), and beta diversity was analyzed using generalized UniFrac distances (Lozupone et al., 2011; Chen et al., 2012). From these distances, non-metric multidimensional scaling (NMDS) was performed and plotted, and a permutational multivariate analysis of variance (PERMANOVA) was used to test for significant differences in community structure using the vegan (Oksanen et al., 2019) and pairwise Adonis (Arbizu, 2017) packages in R. To ensure significant differences were not the result of unequal dispersion of variability between groups, permutational analyses of dispersion (PERMDISP) were conducted for all significant PERMANOVA outcomes using the vegan package. Furthermore, the relative abundances (RAs) of ASVs within each sample were calculated and plotted using phyloseq. Changes in BRSV titer from day 0 to day 28 were calculated with the following equation:


$$\begin{array}{c} \Delta \log_{2}BRSV\;titre=\;Day\;28\;\log_{2}BRSV\;titre\;-Day\;0\;\log_{2}BRSV\;titer\; \end{array}$$

From these values, animals were categorized into three groups for community-wide analysis: no response (log_2_*BRSV titer* ≤ 0), low response (0 < log_2_*BRSV titer*≥4), and high response (5 ≤ log_2_*BRSV titer*≥ 9).

The M:M ratio was calculated with the following equation:


$$\begin{array}{c} M:M\;ratio=\;\frac{Relative\;abundance\;of\;Mycoplasma\;\left(\%\right)}{Relative\;abundance\;of\;Moraxella\;\left(\%\right)}\; \end{array}$$

The RA (%) of both *Mycoplasma* and *Moraxella* was from the same community (i.e., single sample).

### 2.8. Statistical analyses

Unless indicated otherwise, R version 4.1.0 (R Core Team, 2021) was used for the statistical analysis of data. Pairwise Wilcoxon rank-sum tests were performed with a Benjamini-Hochberg correction for multiple comparisons. Differences in beta diversity were tested using pairwise PERMANOVA with a Benjamini-Hochberg correction for multiple comparisons and 9,999 permutations. Additionally, pairwise PERMDISPs were carried out for all significant PERMANOVA outcomes using 9,999 permutations to test for differences in multivariate variation in relation to the comparison of interest. To determine the genera that best explained the change in log_2_(BRSV titer) from day 0 to day 28, a linear model was fitted using all combinations of the log(M:M ratio), treatment group (INJ or INT), and the changes in RA of all genera representing at least 1% of the overall community (*Faucicola, Filobacterium Histophilus, Mannheimia, Moraxella, Mycoplasma*, and *Ureaplasma*) across all samples and the best model was chosen based on corrected Akaike's information criterion (AIC). A Shapiro–Wilk test and quantile–quantile plots were used to test data for normality and Breusch–Pagan tests were used to test for homoscedasticity with an alpha of 0.05. Mean separation was evaluated using a critical alpha level of 0.05.

## 3. Results

### 3.1. Differences in respiratory microbial communities following respiratory vaccine administration

**Figure 1:**
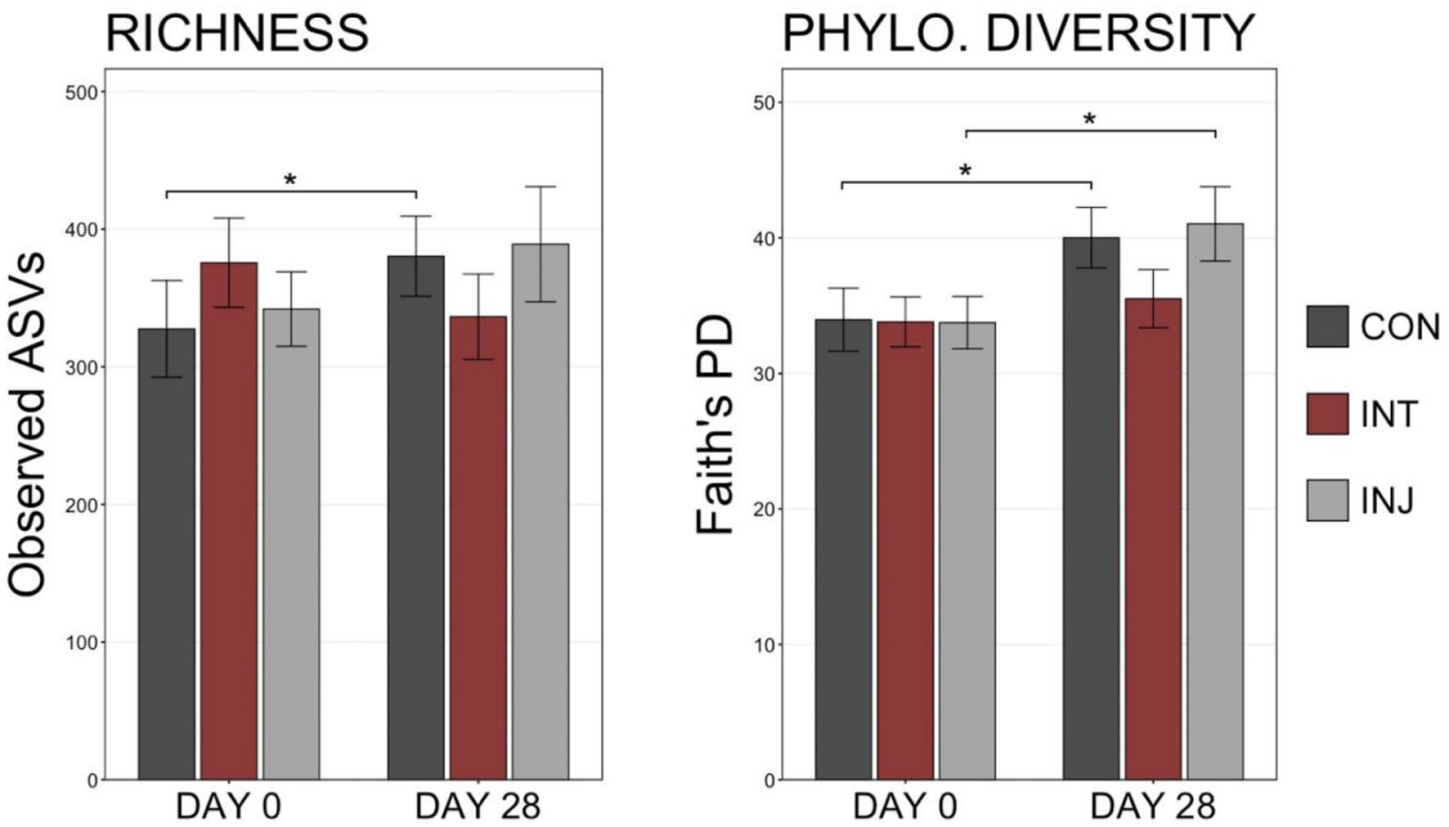
Boxplots displaying the number of observed ASVs and Faith's phylogenetic distance from all samples in each treatment on d 0 and d 28. Significant differences in richness and diversity between components are denoted by * (Pairwise Wilcoxon rank-sum with Benjamini-Hochberg correction, *P* < 0.05).

There were no significant differences in the richness or diversity of respiratory microbiome when comparing animals administered either to the vaccine treatment group (INJ or INT) or to the control group on d 0 or d 28 (Figure 1; pairwise Wilcoxon rank-sum with Benjamini-Hochberg correction, *n* = 138–162, *P* = 0.10). Among animals that remained healthy throughout the study, richness and diversity were not significantly different among the three vaccine treatment groups (INJ, INT, and CON) on d 0 or d 28 (Supplementary Figure 1; pairwise Wilcoxon rank-sum with Benjamini-Hochberg correction, *n* = 69–84, *P* = 0.19). However, richness increased between d 0 and d 28 within the CON group (*P* = 0.01), and diversity increased between d 0 and d 28 in the INJ and CON groups (Supplementary Figure 1; pairwise Wilcoxon rank-sum with Benjamini-Hochberg correction, *n* = 69–84, INJ *P* = 0.04, CON *P* = 0.009). An increase in richness or diversity was not observed (*P* = 0.36) for INT between these dates.

**Figure 2:**
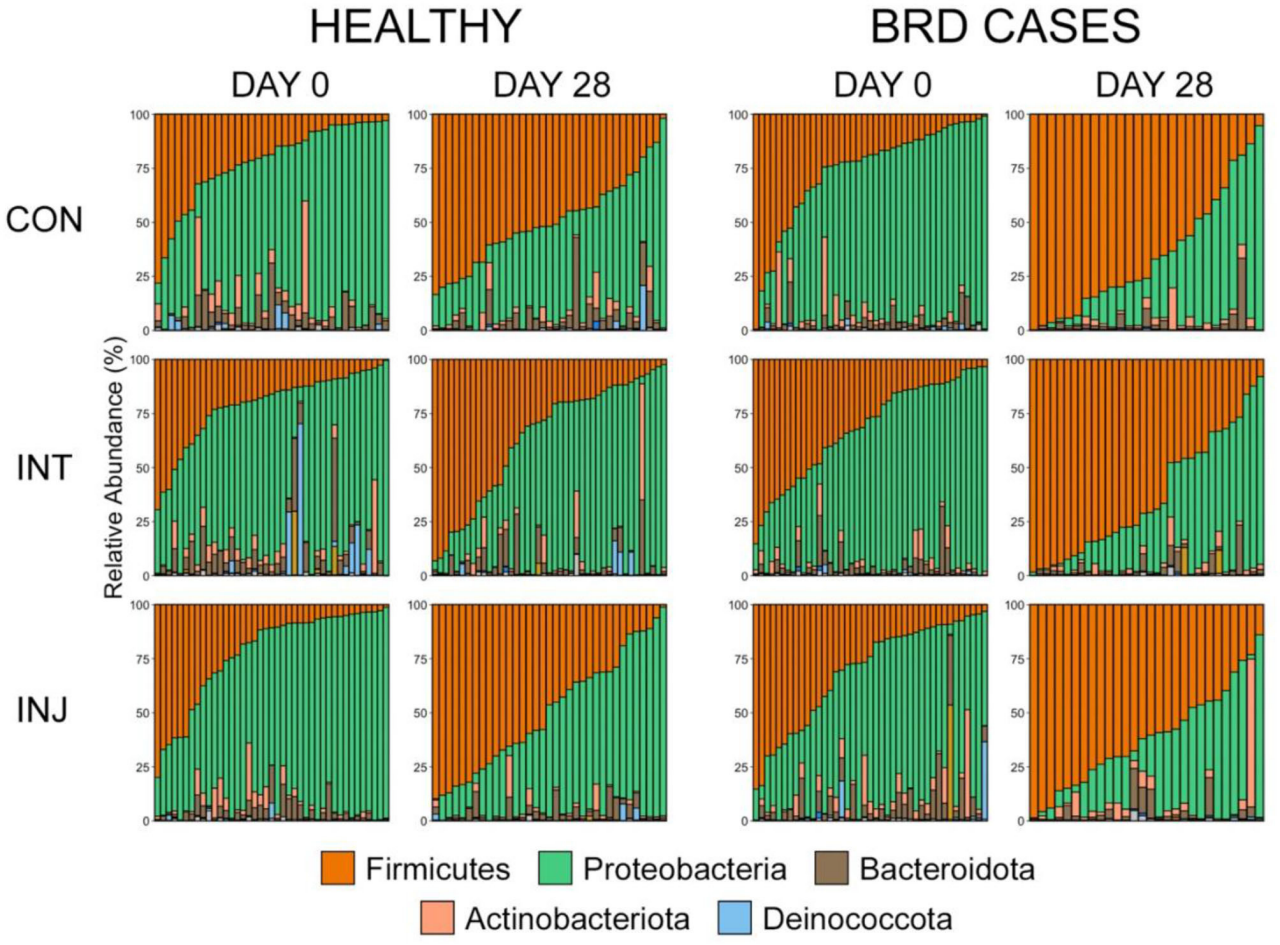
Bar plots showing the relative abundances of the phyla by health classification, day, and treatment. Colors represent the different phyla. The top five most abundant phyla are displayed in the legend.

The microbiome composition did not differ significantly on d 0 or d 28 among the three vaccine treatment groups when analyzing data from all animals enrolled in the study or within animals that remained healthy (Supplementary Table 1, PERMANOVA with Benjamini-Hochberg correction, *n* = 62-85, *P* = 0.06). Ordination with NMDS also illustrated that bacterial communities did not differ among study groups (Supplementary Figure 1). The taxonomic composition of the microbial communities from each treatment group was further examined on d 0 and d 28 using NMDS (Supplementary Figure 1). On d 0, Proteobacteria was the most predominant phylum in all three vaccine treatment groups (mean RA ± SEM, CON = 65.0% ± 2.8; INT = 61.1% ± 2.5; INJ = 61.7% ± 2.7, followed by Firmicutes (mean RA ± SEM, CON = 24.2% ± 2.5; INT = 26.5% ± 2.3; INJ = 26.4% ± 2.7) (Figure 2; Supplementary Table 2). Bacteroidota, Actinobacteria, and Deinococcota were the next most abundant phyla and were similarly represented in all vaccine treatment groups (Figure 2; Supplementary Table 2). Combined, these five phyla represented over 98% of the RA in the overall microbial community in all treatment groups on d 0. By d 28, Firmicutes (mean RA ± SEM, CON = 56.2% ± 3.2; INT = 48.0 ± 3.5; INJ = 54.7% ± 3.2) had the highest RA followed by Proteobacteria (mean RA ± SEM, CON = 35.4% ± 3.0; INT = 43.7% ± 3.5; INJ = 37.2% ± 3.3) within all three vaccine treatment groups. While communities from animals within the INT treatment group had lower RAs of Firmicutes and higher RAs of Proteobacteria on d 28, those differences were not statistically significant (Figure 2; Supplementary Table 2; pairwise Wilcoxon rank-sum with Benjamini-Hochberg correction, *n* = 62–77, *P* = 0.27).

To account for the impact of morbidity on the URT microbiome in differently vaccinated cattle, the rank of phylum was used to visualize the taxonomic composition of the microbial communities from healthy animals on d 28. In that population, Firmicutes (CON = 49.3% ± 3.4; INT = 36.1% ± 4.3; INJ = 49.3% ± 4.6) was the predominant phylum in animals from the CON and INJ group, but Proteobacteria (CON = 40.4% ± 3.4; INT = 53.7% ± 4.7; INJ = 44.0% ± 4.6) was most abundant within INT (Figure 2; Supplementary Table 2). Interestingly, healthy animals on d 28 receiving INT had significantly lower RAs of Firmicutes than those in the CON (*P* = 0.04) and INJ (*p* = 0.05) treatment groups (Figure 2; Supplementary Table 2; pairwise Wilcoxon rank-sum with Benjamini-Hochberg correction, *n* = 35–43).

**Figure 3:**
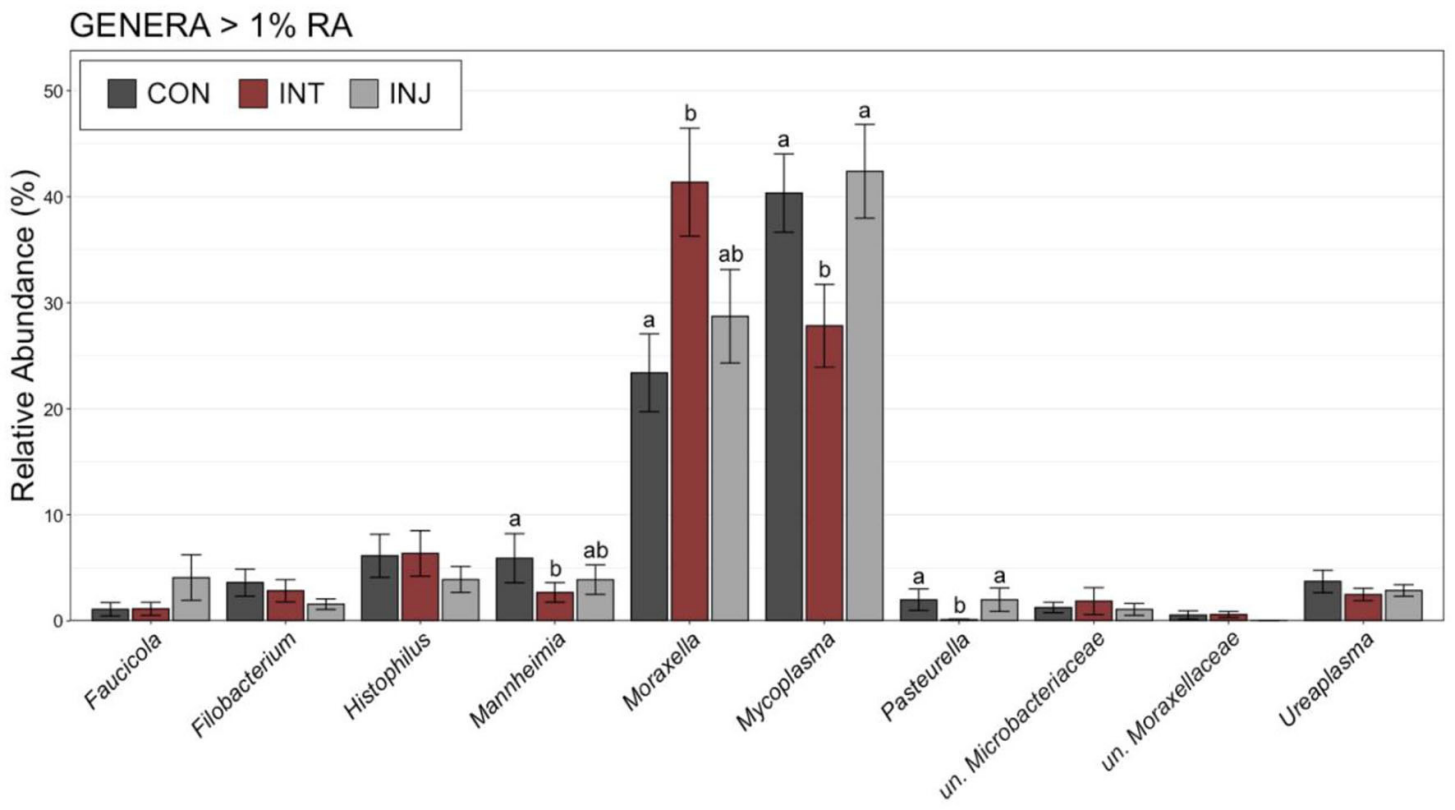
Bar plot showing the relative abundances of the genera with >1% relative abundance of the microbial communities in nasopharyngeal samples collected from healthy cattle on d 28. Error bars display the standard error of the mean for each genus, and colors identify the different treatment groups. The 10 most abundant genera are displayed. Groups with different superscript letters have significantly different mean relative abundances (Pairwise Wilcoxon rank-sum with Benjamini-Hochberg correction, *P* < 0.05).

**Figure 4:**
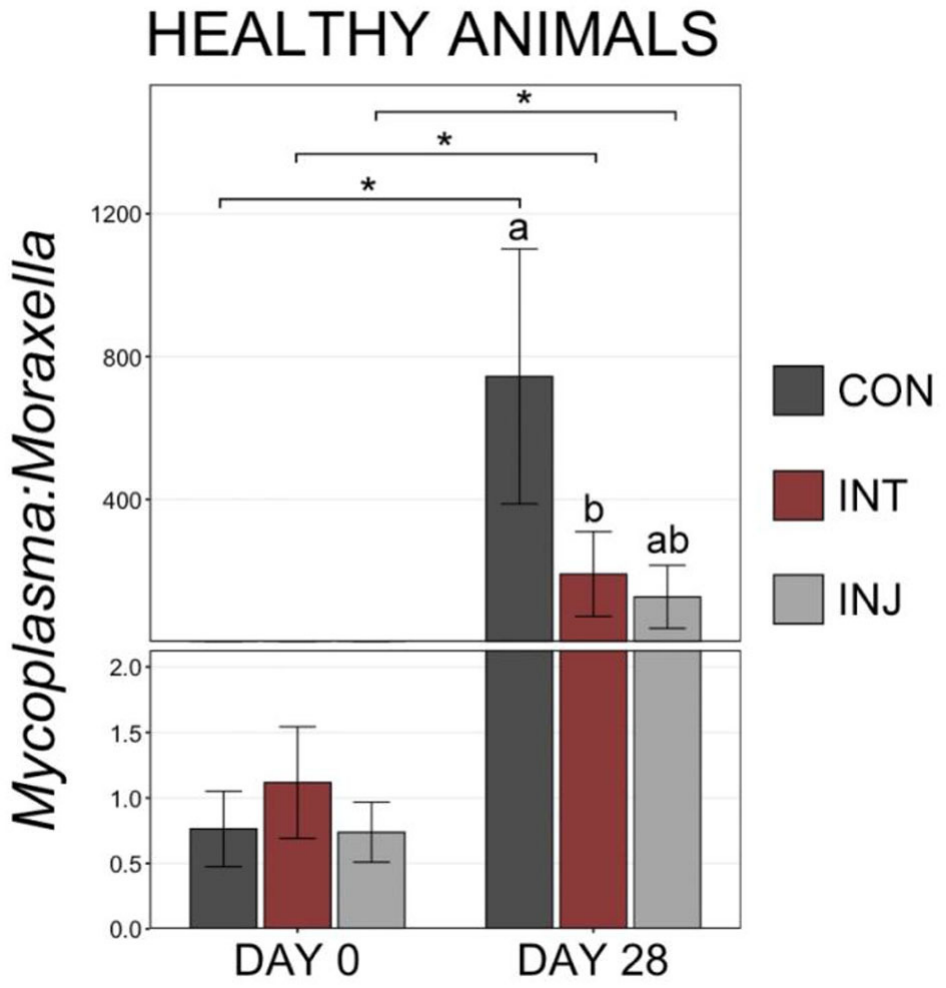
Bar plot showing the ratio of relative abundances for *Mycoplasma* and *Moraxella* genera in samples collected from healthy cattle on d 28. Error bars display the standard error of the mean for each treatment, and colors identify the different treatment groups. Significant differences in the RA of the genera are denoted by * (Pairwise Wilcoxon rank-sum with Benjamini-Hochberg correction, *P* < 0.05).

To examine taxonomic differences at a more granular level among study groups, RAs for genera comprising at least 1% of the overall community were visualized and compared on d 0 and d 28. At d 0, *Moraxella* was the most predominant genus followed by *Mycoplasma*, and combined the two genera comprised over 50% of the microbial community in all vaccine treatment groups (Supplementary Figure 1). There were no significant differences in any of the genera comprising more than 1% of the overall community between the treatment groups at d 0 (Supplementary Figure 1; pairwise Wilcoxon rank-sum with Benjamini-Hochberg correction, *n* = 76–85, *P* = 0.24). Across all animals on d 28, *Mycoplasma* was the most abundant genus followed by *Moraxella* (Supplementary Figure 1). Although *Moraxella* had a greater RA than *Mycoplasma* in the INT group, the differences were not statistically significant (pairwise Wilcoxon rank-sum with Benjamini-Hochberg correction, *n* = 62–77, *P* = 0.20). However, *Histophilus* was in higher RA within INT animals compared to CON and INJ at d 28, while *Mannheimia* was of significantly lower RA for INT compared to CON (Supplementary Figure 1, pairwise Wilcoxon rank-sum with Benjamini-Hochberg correction, *n* = 62–77, *P* = 0.03). To account for potential impacts associated with morbidity, the RAs of genera comprising at least 1% of the overall community were visualized and compared among healthy animals at d 28. In healthy animals, *Moraxella* was the most abundant genus within the INT group, while *Mycoplasma* was the most abundant in both CON and INJ animals (Figure 3). *Moraxella* was also more abundant in healthy INT animals compared to healthy CON animals (*P* = 0.05), and *Mycoplasma* was significantly less abundant in healthy INT animals compared to healthy CON and INJ on d 28 (Figure 3; pairwise Wilcoxon rank-sum with Benjamini-Hochberg correction, *n* = 62–77, *P* = 0.02). Furthermore, *Mannheimia* was in significantly lower RA in healthy INT animals compared to healthy CON animals, and *Pasteurella* was in significantly lower RA in healthy INT animals compared to both healthy CON and healthy INJ animals on d 28 (Figure 3; pairwise Wilcoxon rank-sum with Benjamini-Hochberg correction, *n* = 62–77, *P* < 0.02). Given the overwhelming predominance of *Mycoplasma* and *Moraxella*, and the importance of *Mycoplasma* in BRD, a ratio of *Mycoplasma:Moraxella* RAs (M:M ratio) was calculated and compared between vaccine treatment groups in healthy animals at d 0 and 28. Animals in the INT group had significantly lower M:M than the CON group at d 28 (*P* = 0.03), but the M:M ratio increased in all vaccine treatment groups over time (Figure 4; pairwise Wilcoxon rank-sum with Benjamini-Hochberg correction, *n* = 62–77, INT *P* = 0.007, CON *P* < 0.001, INJ *P* < 0.001).

### 3.2. Relationship with BRSV antibody response

**Figure 5:**
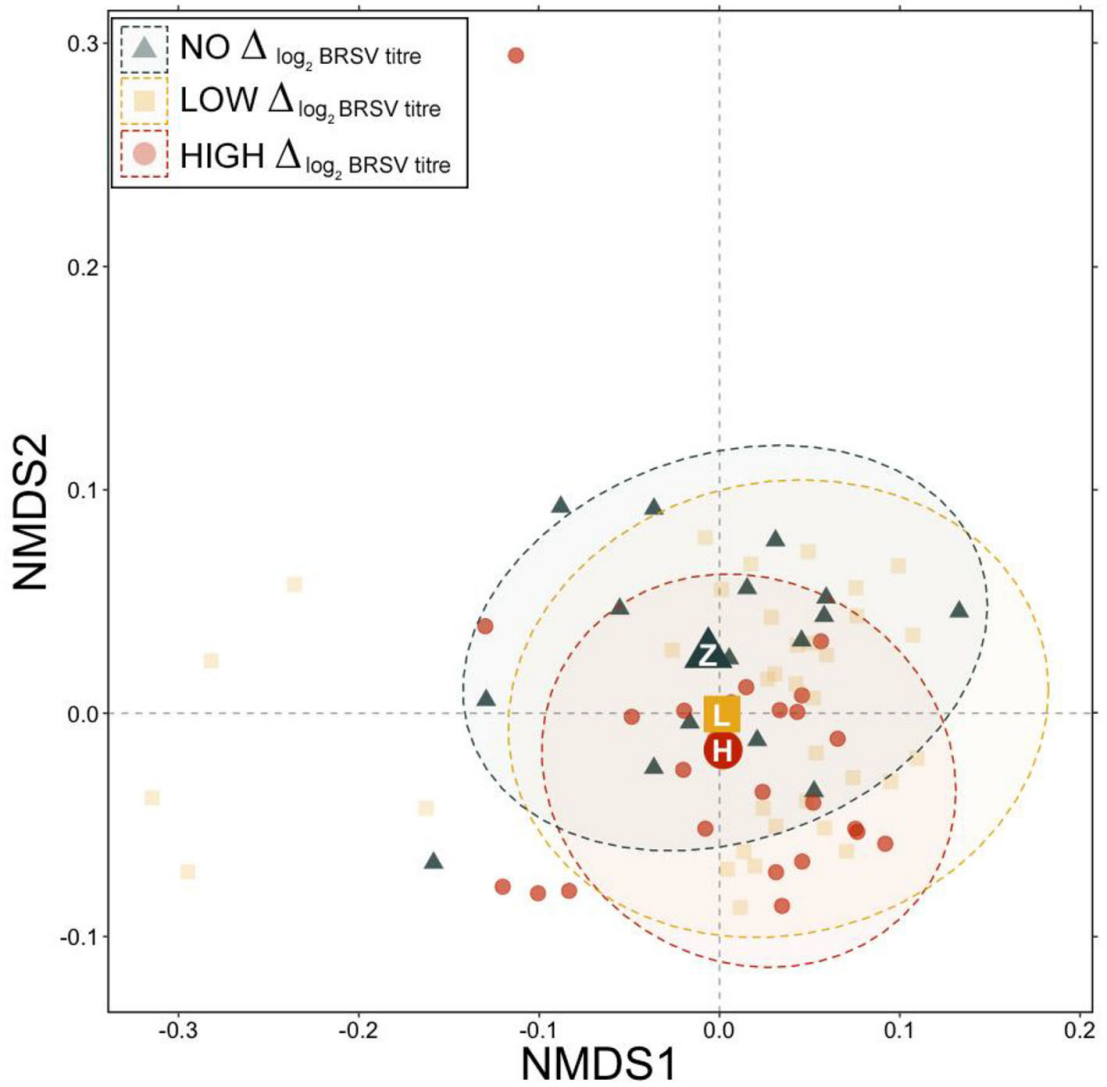
Non-metric multidimensional scaling (NMDS) of generalized UniFrac distances illustrating variation in microbial community structure associated with different BRSV antibody responses. The NMDS demonstrates clustering of 16S rRNA gene sequences from animals classified as having no response, low response, or high response at d 28. The large, opaque point represented the centroid of that group, with more transparent points representing individual samples. Dashed lines and shaded areas represent 95% confidence ellipses for each group.

There were no significant differences in the RA of any of the nine dominant genera between animals that were seropositive vs. seronegative to BSRV on d 28 (Supplementary Figure 3; pairwise Wilcoxon rank-sum with Benjamini-Hochberg correction, *n* = 9–67, *P* = 0.46). However, based on generalized UniFrac distances, a significant difference in day 28 community structure was detected between communities from animals that had no change in log_2_ BRSV titer vs. those classified as high responders (Figure 5, PERMANOVA with Benjamini-Hochberg correction, *n* = 16–25, *P* = 0.03). The change in BRSV titer from d 0 to d 28 was best explained by changes in the RA of *Mycoplasma* (Table 2; R^2^ = 0.088; *P* = 0.006). Differences were notable in average RA for *Mycoplasma* and *Moraxella* between animals that had a high vs low log_2_ BRSV titre change, but the variability in responses was quite large and these differences were not statistically significant (*P* < 0.05, Supplementary Figure 4). Interestingly, no combination of other abundant genera (*Faucicola, Filobacterium, Histophilus, Mannheimia, Moraxella*, or *Ureaplasma*), the log M:M ratio, or vaccine treatment were significantly associated with differences in the BRSV antibody response categories.

**Table 2:** Summary of multiple linear regression investigating microbiome features associated with changes in BRSV serum titers from day 0 to day 28.

	**Estimate**	**Std. Err**.	***t*-value**	***P***
Intercept	2.779	0.321	8.66	9.98^−13^
*Mycoplasma*	0.022	0.007	2.82	0.006
F-statistic = 7.952 on 1 and 71 d.f.; adjusted R^2^ = 0.088; overall model *p* = 0.006				

Exposure variables investigated included changes in between d 0 and d 28 in the RA (RA) of predominant genera (*Mycoplasma, Moraxella, Mannheimia, Faucicola, Pasteurella, Histophilus, Ureaplasma*, and *Filobacterium*), the log M:M ratio at day 28, and treatment group (INJ or INT). The best model was identified comparing all possible variable combinations, and the final model only included changes in the RA of *Mycoplasma*. Std. Err., standard error; d.f., degrees of freedom; M:M, *Mycoplasma:Moraxella*.

### 3.3. Differences in microbial communities based on health status

**Figure 6:**
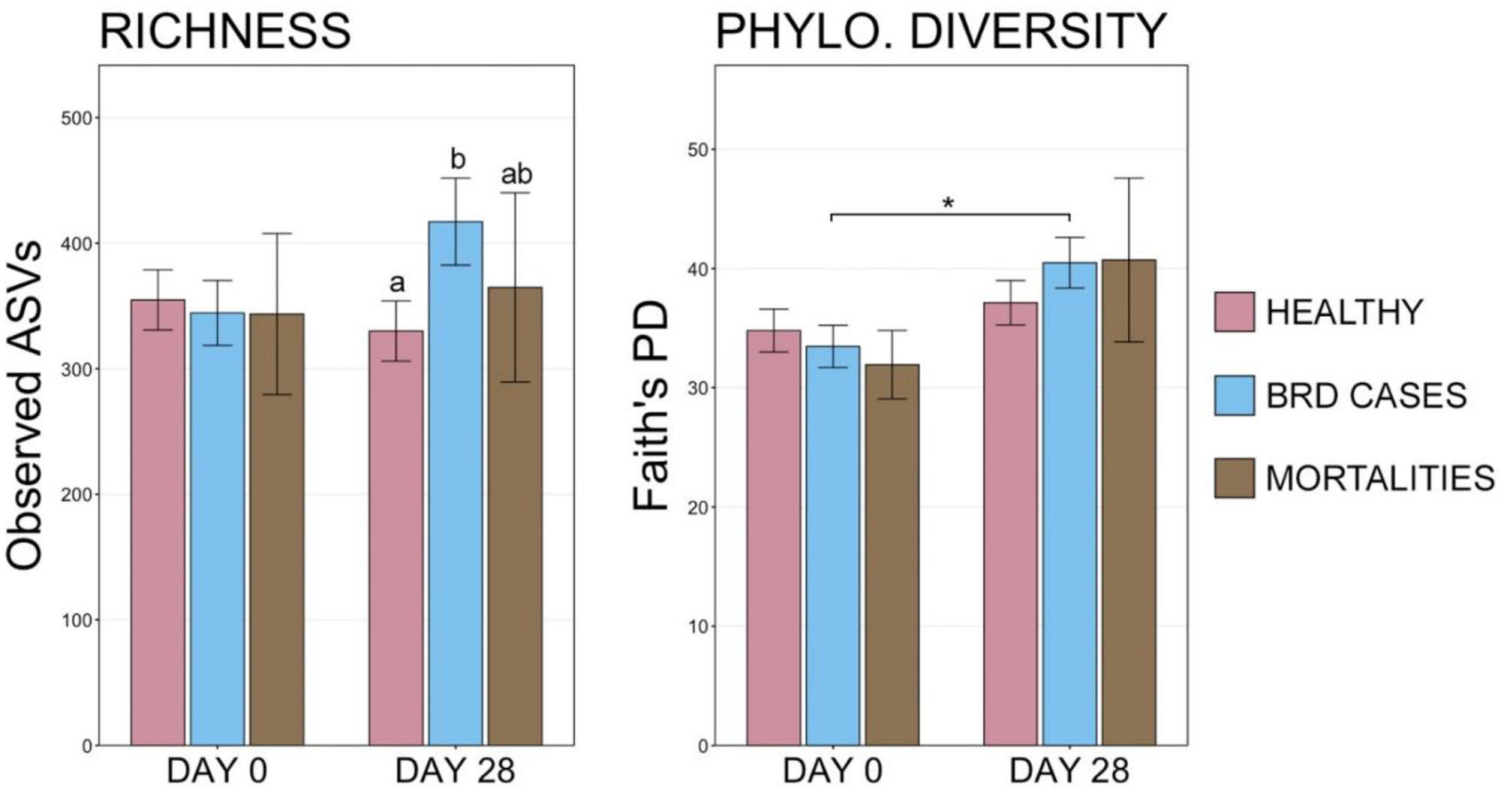
Bar plots displaying the number of observed ASVs and Faith's phylogenetic distance from final health classification on d 0 and d 28. Significant differences in richness and diversity at a timepoint are designated by groups with different superscript letters, and significant differences between sampling days are denoted with * (Pairwise Wilcoxon rank-sum with Benjamini-Hochberg correction, *P* < 0.05).

At d 0, richness and diversity were similar among animals with different final health outcomes. However, at d 28, BRD cases had richer communities than healthy animals, though only eight animals that died during the study period could be sampled at d 28 (Figure 6, pairwise Wilcoxon rank-sum with Benjamini-Hochberg correction, *n* = 8–113, *P* = 0.04). Diversity also increased significantly over time in BRD-affected animals but not in healthy animals or mortalities (Figure 6; pairwise Wilcoxon rank-sum with Benjamini-Hochberg correction, *n* = 8–113, *P* = 0.01). Similarly, the overall composition of microbial communities on d 0 did not differ among cattle in different health classification groups, but community composition between BRD cases and healthy animals differed significantly on d 28 (Supplementary Table 1, PERMANOVA with Benjamini-Hochberg correction, *n* = 8–113, *P* = 0.001). Visualization with NMDS illustrated that microbial communities in d 0 samples did not differ based on final health classification, but there was a subtle divergence in community structures between healthy animals and BRD cases on d 28 (Supplementary Figure 2).

Despite the similarity in overall microbial community structures, cattle that became BRD cases had an increased RA of Firmicutes on d 0 compared to cattle that remained healthy (Figure 2; pairwise Wilcoxon rank-sum with Benjamini-Hochberg correction, *n* = 42–116, *P* = 0.04), but there were no significant differences associated with other predominant phyla. On d 28, BRD cases had higher RA of Firmicutes and lower RA of Proteobacteria than cattle that remained healthy (Figure 2; pairwise Wilcoxon rank-sum with Benjamini-Hochberg correction, *n* = 8–113, Firmicutes *P* < 0.001, Proteobacteria *P* < 0.001). At the rank of family, the RA of Mycoplasmataceae increased from d 0 to d 28, particularly among BRD cases (Supplementary Figure 2).

**Figure 7:**
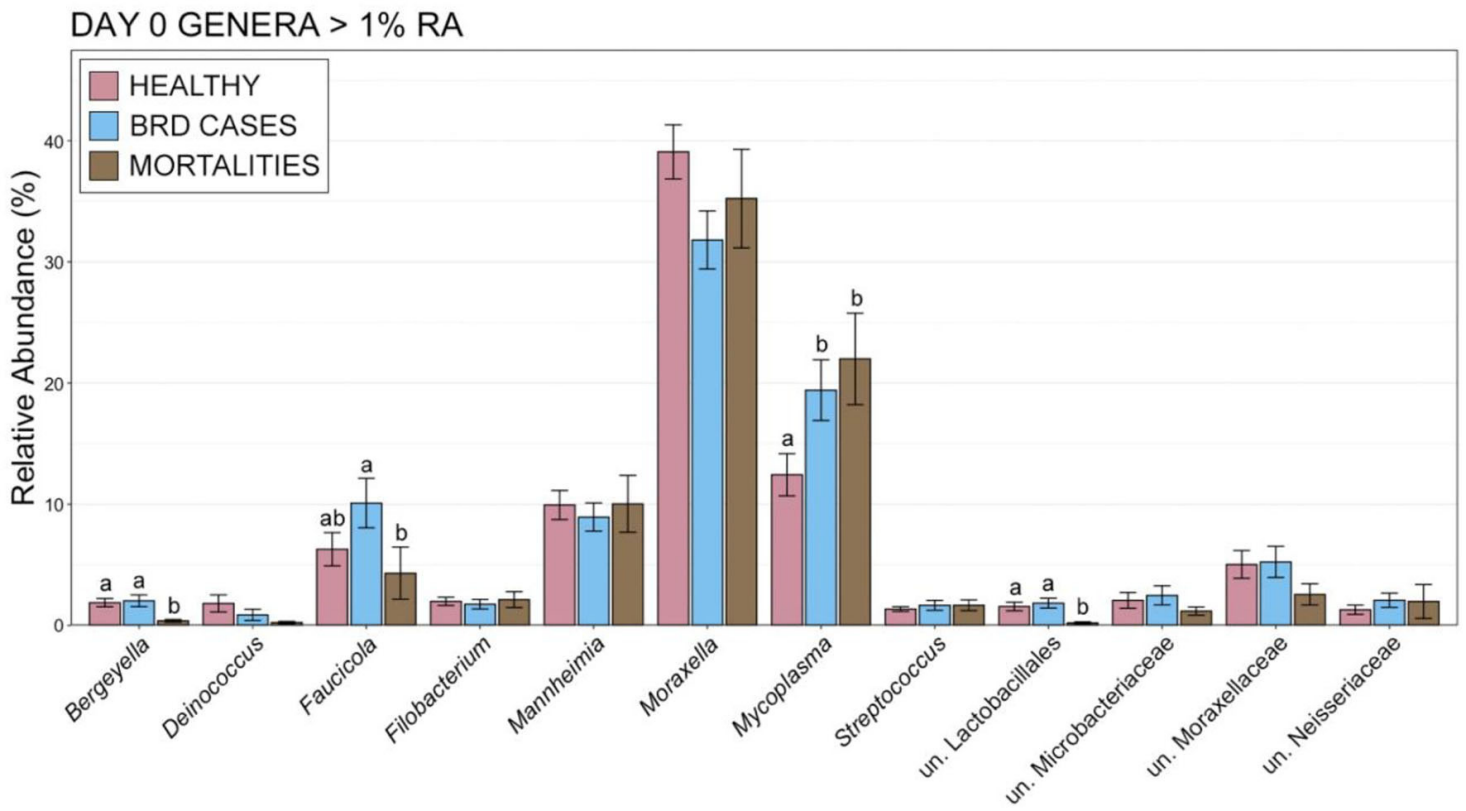
Bar plot showing the relative abundances within the nasopharyngeal microbial communities on d 0 for genera with >1% relative abundance. Error bars display the standard error of the mean, and colors identify cattle with different health statuses at the end of the feeding period. The 12 most abundant genus are displayed. Groups with different superscript letters have significantly different mean relative abundances (Pairwise Wilcoxon rank-sum with Benjamini-Hochberg correction, *P* < 0.02).

The increased abundance of Firmicutes was largely the result of an increased RA of the genus *Mycoplasma*, which was more abundant on d 0 within animals that became BRD cases or died during the 70-day study (Figure 7; pairwise Wilcoxon rank-sum with Benjamini-Hochberg correction, *n* = 42–116, *P* = 0.04). Other important BRD pathogens including *Mannheimia, Pasteurella*, and *Histophilus* either represented < 1% of the overall community or did not differ on d 0 (Figure 7; pairwise Wilcoxon rank-sum with Benjamini-Hochberg correction, *n* = 42–116, *P* = 0.76) among healthy cattle, BRD cases, and animals that died. At d 28, BRD cases and individuals that died had higher RAs of *Mycoplasma* than healthy cattle (Supplementary Figure 5; pairwise Wilcoxon rank-sum with Benjamini-Hochberg correction, *n* = 8–113, *P* < 0.001). Animals that remained healthy also had higher RAs of *Moraxella* than BRD cases at d 28 (Supplementary Figure 5; pairwise Wilcoxon rank-sum with Benjamini-Hochberg correction, *n* = 8–113, *P* < 0.001). Higher RA of *Mycoplasma* and lower RA of *Moraxella* was also seen for BRD Cases with fevers (CIS = 3) compared to those that did not have fevers (CIS = 2; Supplementary Figure 6). The RA of other important BRD pathogens such as *Mannheimia, Pasteurella*, and *Histophilus* did not differ on d 28 between any of the health categories (Supplementary Figures 5, 6; pairwise Wilcoxon rank-sum with Benjamini-Hochberg correction, *n* = 8–113, *P* = 0.17).

**Figure 8:**
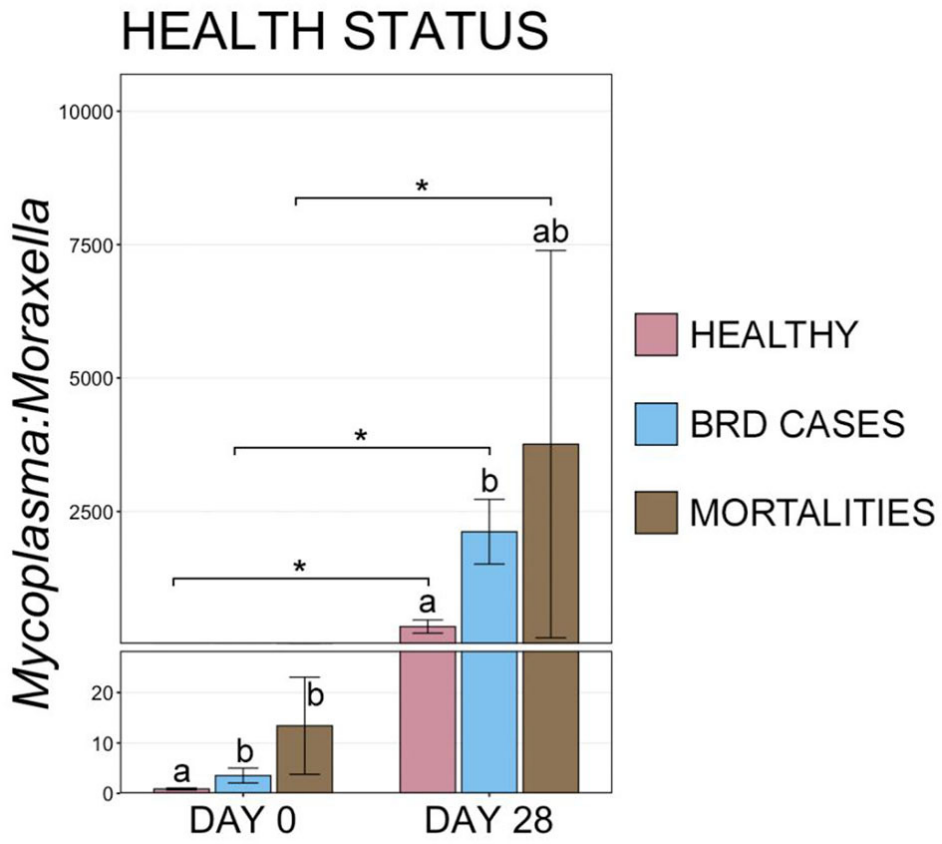
Bar plot showing the ratio of relative abundances for *Mycoplasma:Moraxella* genera, by final health classification. Error bars display the standard error of the mean, and cattle with different health classifications are denoted by different colors. Significant differences in the M:M ratio at a single timepoint are identified by different superscript letters, and significant differences between timepoints are denoted with * (Pairwise Wilcoxon rank-sum with Benjamini-Hochberg correction, *P* < 0.05).

Interestingly, at d 0, animals that remained healthy throughout the study had a lower M:M ratio than animals that became a BRD case or died (Figure 8, pairwise Wilcoxon rank-sum with Benjamini-Hochberg correction, *n* = 42–116, *P* = 0.03). At d 28, healthy animals continued to have a lower M:M ratio than BRD cases, but small sample size and large variation in M:M ratios within the remaining cattle that would go on to die resulted in a lack of statistical difference for that group (Figure 8, pairwise Wilcoxon rank-sum with Benjamini-Hochberg correction, *n* = 8–113, *P* > 0.001).

Samples collected at the time of the first BRD treatment had the greatest RA of Firmicutes (BRD = 60.3% ± 2.6; d 0 = 25.7% ± 1.4; d 28 = 52.6% ± 1.6) at the phylum level and *Mycoplasma* (BRD = 53.1% ± 2.5; d 0 = 16.5% ± 1.4; d 28 = 43.7% ± 1.90) at the genus level that exceeded abundance at BRD treatment than at d 0 or d 28 (Supplementary Tables 3–5; pairwise Wilcoxon rank-sum with Benjamini-Hochberg correction, *n* = 114–243, *P* < 0.05). At the time of initial BRD treatment, the RAs of Proteobacteria (BRD = 37.1% ± 2.6; d 0 = 62.5% ± 1.6; d 28 = 39.1% ± 1.9) and *Moraxella* (BRD = 25.6% ± 2.1; d 0 = 35.9% ± 1.5; d 28 = 26.1% ± 2.0) were both significantly lower than at d 0 but did not differ from d 28 (Supplementary Tables 3–5; pairwise Wilcoxon rank-sum with Benjamini-Hochberg correction, *n* = 114 to 243, *P* < 0.05).

### 3.4. Temporal shifts in URT community composition from d 0 to d 28

To examine potential temporal shifts in the microbiome over the first 28 d in the feedlot, the composition of URT microbial communities was compared between d 0 and d 28 for animals within each health category (i.e., healthy, BRD cases, and mortalities). Ordination with NMDS illustrated that communities clustered by timepoint (i.e., d 0 vs. d 28) for animals in all three health categories, suggesting there was a shift in URT community composition over this time, independent of vaccination or health status, and PERMANOVA confirmed this shift was significant (Figure 8, Supplementary Table 1, *n* = 8–116, healthy *P* = 0.001, BRD cases *P* = 0.001, mortalities *P* = 0.002).

**Figure 9:**
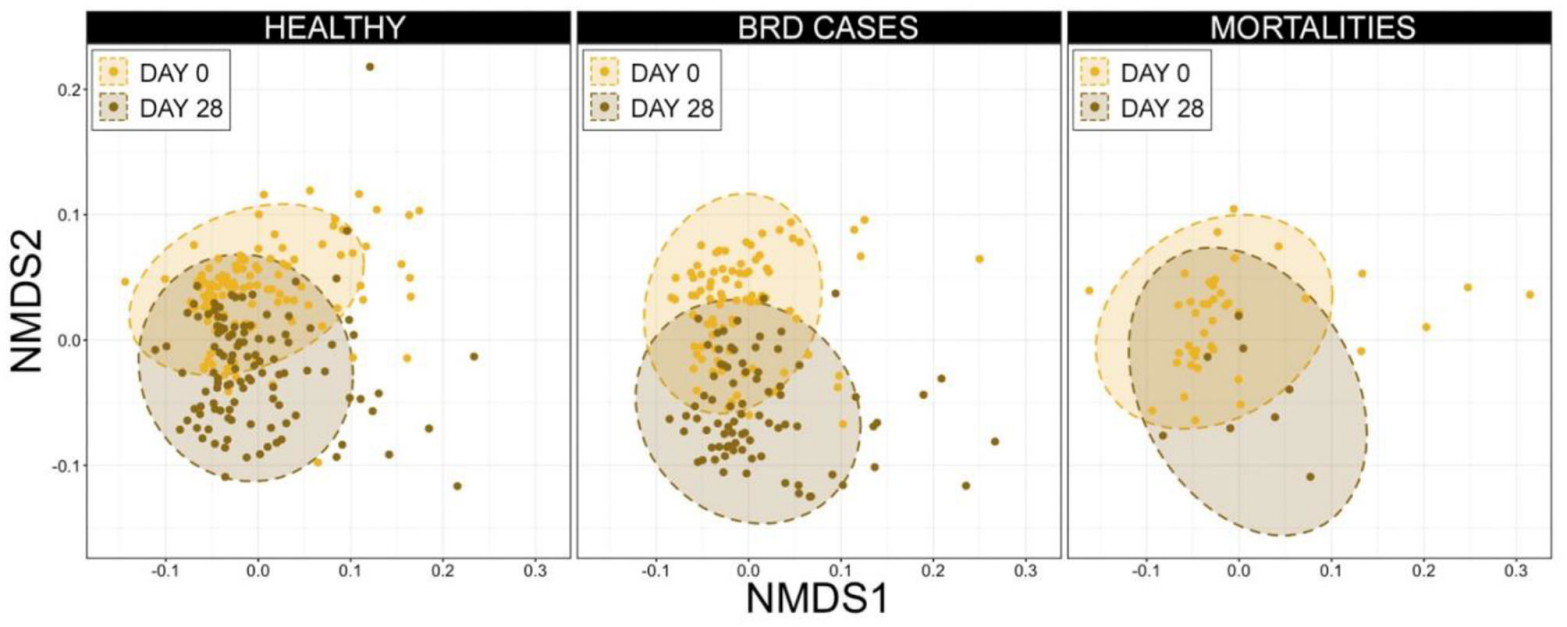
Non-metric multidimensional scaling (NMDS) generalized UniFrac distances illustrating variation in microbial community structures, by day and final health status. These data demonstrate clustering (i.e., similarity and changes over time) among microbial community structure from d 0 to d 28. Dashed lines and shaded areas represent 95% confidence ellipses for each day.

**Figure 10:**
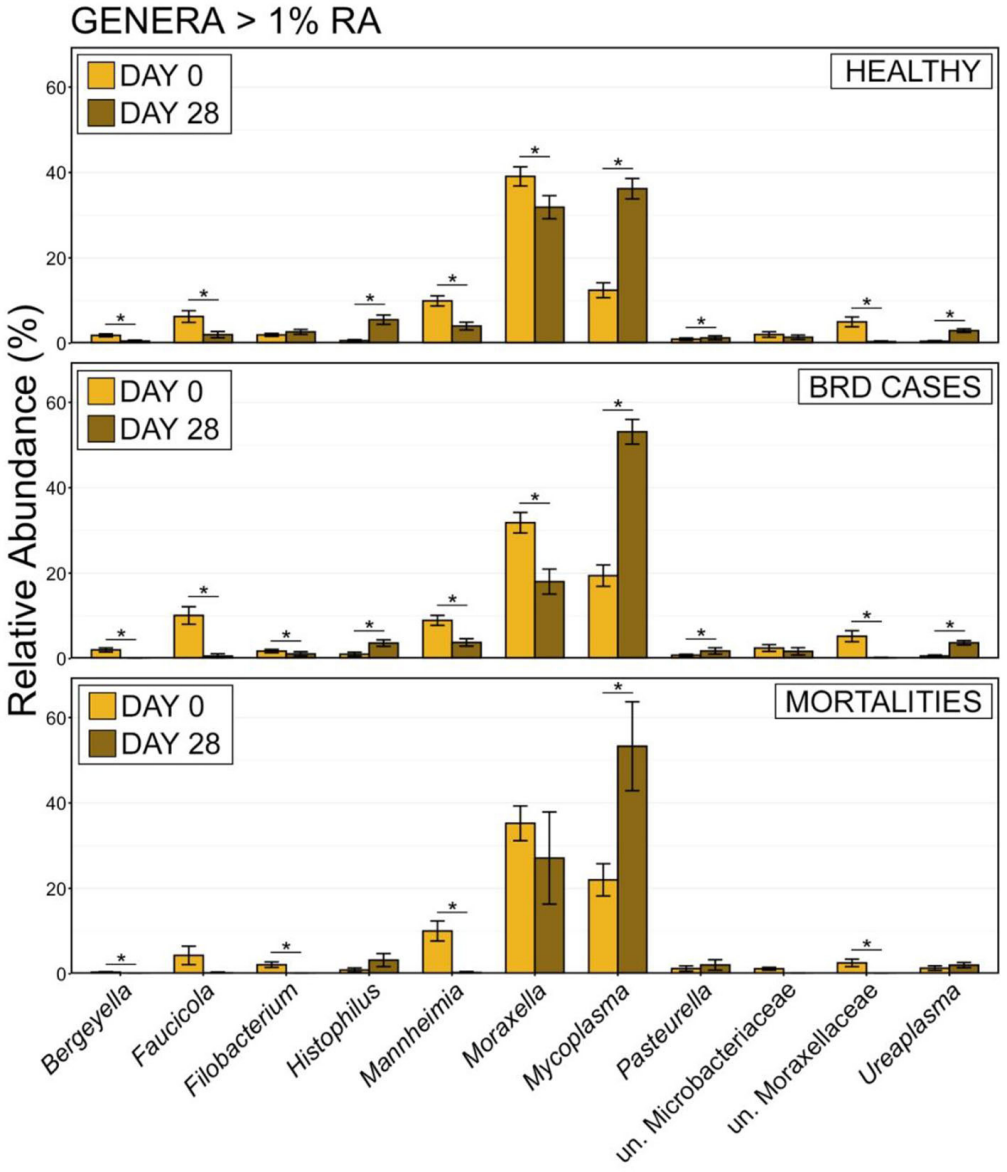
Bar plot showing the relative abundances of the genera of >1% relative abundance of the total community from all samples by day. Error bars display the standard error of the mean. Sampling timepoint (d 0 or d 28) are identified by bar color. The 11 most abundant genera are displayed in the legend. Significant differences in the relative abundance between sampling days are denoted by * (Pairwise Wilcoxon rank-sum with Benjamini-Hochberg correction, *P* < 0.05).

In addition to shifts in the overall composition, the RA of different phyla also changed between d 0 and d 28. The most predominant phylum, Firmicutes, doubled in mean RA from 25.71% ± 1.41 (mean RA ± SEM) at d 0 to 52.60% ± 1.95 at d 28, while the second most abundant phylum Proteobacteria significantly decreased in RA from 62.53% ± 1.55 on d 0 to 39.12 ± 1.93 on d 28 (Supplementary Table 3; pairwise Wilcoxon rank-sum with Benjamini-Hochberg correction, *n* = 202–243, *P* < 0.001). To investigate these temporal shifts further, differences in the Ras of genera comprising at least 1% of microbial communities were compared between samples collected at d 0 vs. d 28 for each health status. There were significant shifts in the RAs of all but 2 of the 11 most abundant genera in cattle that remained healthy and those that developed BRD (Figure 9). In both health status groups, *Mycoplasma, Histophilus, Pasteurella*, and *Ureaplasma* all significantly increased in RA from d 0 to d 28, while *Moraxella, Mannheimia, Faucicola, Bergeyella*, and unclassified members of Moraxellaceae all decreased (Figure 10; pairwise Wilcoxon rank-sum with Benjamini-Hochberg correction, *n* = 202–243, *P* < 0.05). While similar trends were observed among cattle that died, owing to small samples sizes and large variation at d 28, only the increase in *Mycoplasma*, and the decrease in *Mannheimia* and unclassified members of Moraxellaceae were statistically significant in cattle that died during the study (Figure 9; pairwise Wilcoxon rank-sum with Benjamini-Hochberg correction, *n* = 202–243, *P* < 0.05).

## 4. Discussion

Using 16S rRNA gene sequencing, this study found that the URT microbiome differed in cattle that were vaccinated intra-nasally with MLV targeting respiratory viruses compared with cattle that were vaccinated parenterally against the same viruses or unvaccinated CON cattle. However, changes in serum antibody titers to BRSV were not associated with differences in URT microbiome composition. Together, these findings suggest that changes in URT microbial communities were mediated by local immunity or interactions among microbial features within the respiratory tract as opposed to being mediated by systemic immune responses. Perhaps it is most notable that cattle vaccinated intra-nasally had lower RAs of *Mycoplasma* spp. post-vaccination, given the differences in RAs of *Mycoplasma* and *Moraxella* that were evident among cattle with different health statuses (i.e., healthy, BRD cases, and mortalities). These differences in M:M ratio were also evident at d 0 in cattle that developed BRD or died, suggesting that these taxa may play an important role in the development of BRD or may be important biomarkers that could be used in the prediction of health outcomes for BRD to better target interventions, such as the use of antimicrobial metaphylaxis. Regardless of vaccination or health status, the URT microbial community also demonstrated a clear temporal shift in composition between d 0 and d 28 at the feedlot.

A previous study that evaluated the effects of administering a live-attenuated intranasal influenza virus vaccine in humans reported significant differences in the URT microbial community structures, which was attributed to the vaccine (Tarabichi et al., 2015). Here, intranasal vaccination was associated with a decreased abundance of *Mycoplasma*, which was also observed in a different subset of these same cattle when analyzed using rtPCR (Valeris-Chacin et al., 2022). Yet, Powledge et al. (2022) observed no difference in *Mycoplasma bovis* identified by qPCR among different vaccine treatment groups at any timepoint. Though the overall population of the cattle was the same, a different, but overlapping, subset was selected for sampling in the current study. Additionally, cattle that were positive for any of the BRD pathogens evaluated *via* qPCR on d 0 (BRSV, Hs, *Mycoplasma bovis, Mannheimia haemolytica*, and *Pasteurella multocida*) were excluded from subsequent analysis in Powledge et al. (2022), and this probably contributed to the different results. Furthermore, 16S rRNA gene sequencing detects all taxonomic DNA information from the sample and does not evaluate the species level, only the genus. Intranasal vaccination reduced the abundance of other significant respiratory pathogens (*Mannheimia* and *Pasteurella*). A reduction in these well-known BRD pathogens suggests that INT vaccination may have impacted the respiratory microbiome beneficially. Yet, Powledge et al. (2022) demonstrated no difference between these pathogens among vaccine treatment groups. Conversely, members of *Moraxella*, which are associated with otitis and other infections in cattle (Lima et al., 2016), were increased following INT vaccination.

Intranasal vaccination has been reported to be associated with changes in the respiratory microbiome in humans, mice, and pigs (Mina et al., 2014; Tarabichi et al., 2015; Gierse et al., 2021). Research in humans suggests that viral manipulation or regulation of bacterial community composition in the URT may facilitate the transition of agents from pathobiont to pathogen function (Hanada et al., 2018) and that the enrichment of viral pathways in the URT resulted in specific bacterial taxa impacting host immune responses (Sonawane et al., 2019). Our study represents the first examination of the impacts of MLV vaccination on URT bacterial communities using sequencing-based metagenomic approaches. Additional research is needed to understand the impacts of vaccination, and the route of vaccine administration, on viral infection and replication, and in turn those impacts on microbial composition in the respiratory tract of cattle. Future research should also investigate the absolute abundance of potentially important pathobionts (i.e., *Mycoplasma, Mannheimia, Pasteurella*, and *Histophilus*) within the perspective of entire URT microbial communities.

Health status, specifically the development of clinical BRD, was associated with differences in URT microbiome structure. Importantly, the results of this study suggest that by d 28, there were differences in both overall URT microbial community composition and the RAs of BRD-causing pathogens in animals that remained healthy through d 70 vs. those that developed BRD. These observations are supported by findings in other research suggesting that the respiratory microbiome of cattle differs in animals that develop BRD (Holman et al., 2015; Stroebel et al., 2018; Timsit et al., 2018; Amat et al., 2019; Zeineldin et al., 2020; Crosby et al., 2022). Further research is needed to investigate whether these changes are simply predictive vs. causally associated with BRD occurrence. Sequencing-based metagenomic investigations provide unique insight into complex interactions within entire microbial communities and may provide novel perspectives on BRD pathogenesis and the pathobiont role of otherwise commensal bacteria.

Powledge et al. (2022) found an increase in *Hs* carriage for INT cattle, agreeing with the results of this study and the Gershwin–Corbeil model (Gershwin et al., 2005; Corbeil, 2007). There appears to be an interesting interaction between vaccine type, BRSV antibody, and *Histophilus* spp. abundance. Similarly, *Mycoplasma* spp. was found at a considerably higher RA than either *Mannheimia* spp. or *Histophilus* spp. at the time BRD Cases were first treated. In this study, cattle that remained healthy during the feeding period had lower RA at the genus level for *Mycoplasma* and higher RA of *Moraxella* compared to cattle that developed BRD. *Moraxella* has been reported as one of the most abundant genera in the URT (Stroebel et al., 2018; Zeineldin et al., 2019; McMullen et al., 2020), and *Mycoplasma* and *Moraxella* are found in healthy cattle (Holman et al., 2015). Although *Moraxella* has been implicated as a pathogen associated with respiratory disease and otitis in young calves and is associated with keratoconjunctivitis cattle of all ages, it is more likely to be considered a commensal organism when found in the URT of older cattle. In humans, the phyla Firmicutes and Bacteroidetes dominate in the gut microbiome and represent over 90% of those microbial communities (Qin et al., 2010). The ratio of Firmicutes:Bacteroidetes abundances has been associated with multiple pathological conditions including obesity and inflammatory bowel disease (Magne et al., 2020; Stojanov et al., 2020). Similarly, Mycoplasma and Moraxella are highly abundant in the URT microbiome of cattle, and the M:M ratio may be a useful biomarker for cattle that are likely to develop BRD. Further work is necessary to demonstrate the repeatability of this finding and to clearly elucidate the role of both *Mycoplasma* and *Moraxella* in the development of BRD and other infections such as keratoconjunctivitis.

Previous studies have suggested that cattle with a stable microbial community in the nasopharynx may be at reduced risk of infection from BRD-associated pathogens (Amat et al., 2019). However, our results in high-risk cattle suggest those that remain healthy and those that develop BRD both experience a shift in the URT microbial community composition after feedlot entry. This temporal shift has been demonstrated in both the respiratory and fecal microbiome of feedlot cattle. A limitation of the current study was that the final sampling date of d 28 was too early to determine whether the microbiota had stabilized, as Holman et al. (2015) determined on d 60 that the nasopharyngeal microbiota became more stable. In conclusion, this study provides important insights into the impact of MLV respiratory vaccination on the upper respiratory tract microbiome of cattle through intranasal and parenteral administration routes. There was a significant temporal shift in the URT microbiome composition over the first 28 d in the feedlot, in addition to a shift associated with health status during the same period. Furthermore, BRD may have caused alteration of the URT microbiome and administration of INT, but not INJ, influenced microbiome structure. Further research in larger populations from different environments may confirm this relationship and help explain the dynamics of the respiratory microbiota shift observed in cattle affected by BRD after feedlot arrival.

## Data availability statement

The datasets presented in this study can be found in online repositories. The names of the repository/repositories and accession number(s) can be found below: https://www.ncbi.nlm.nih.gov/, PRJNA898995.

## Ethics statement

The animal study was reviewed and approved by West Texas A&M University Animal Care and Use Committee.

## Author contributions

JR and PM conceived and oversaw the research and provided internal research funding. JR, TM, and SP oversaw cattle management, experimental treatments, and sample collection. PM and CW oversaw and directed all laboratory activities. TM and SP conducted laboratory procedures to purify DNA and prepare amplicon sequencing libraries. LP, TM, PM, and JR planned bioinformatics and statistical analyses and were responsible for the interpretation of results and manuscript planning. TM and LP conducted statistical analyses. TM prepared initial drafts of the manuscript. All authors reviewed, edited, and approved the final manuscript version.

## Supplementary material

Supplementary Figure 1 **(A)** Boxplots displaying the number of observed ASVs and Faith's phylogenetic distance for samples from healthy cattle, by treatment and sampling day. Significant differences in richness and diversity between components are illustrated by different letters (Pairwise Wilcoxon rank-sum with Benjamini-Hochberg correction, *P* > 0.05). **(B)** Non-metric multidimensional scaling (NMDS) generalized UniFrac distances illustrating variation in microbial community structure associated with each treatment. The NMDS demonstrates clustering of *16S rRNA* gene sequences by treatment in all animals. Dashed lines and shaded areas represent 95% confidence ellipses for each treatment. No significant difference in community structure was detected (pairwise PERMANOVA, *P* > 0.05). **(C)** Bar plot showing the RAs of the genera of >1% RA of the total community from all samples by treatment. Error bars display the standard error of the mean for each genera, and colors represent the treatment. The 10 most abundant genera are displayed in the legend. No significant differences in the RA of the genera were found.

Supplementary Figure 2 Non-metric multidimensional scaling (NMDS) generalized UniFrac distances illustrating variation in microbial community structure associated d 0, stratified on final health classification. The NMDS demonstrates clustering of 16S rRNA gene sequences by health status among all animals. Dashed lines and shaded areas represent 95% confidence ellipses for each final health classification category. Bar plots showing the RAs of taxa found in the respiratory microbiota at the family level (RA > 1%) on d 0 and d 28, stratified on final health classification. Colors represent the different family taxa. The eight most abundant families are identified in the legend.

Supplementary Figure 3 Bar plot showing the average RAs of the genera with >1% RA of the total community from d 28 swabs, by vaccination group (INT vs. INJ) and serum BRSV titer status (seronegative vs. seropositive) on d 28. BRSV titer response for INJ and INT. Error bars display the standard error of the mean for each genera and colors represent the BRSV response. The nine most abundant genera are displayed in the legend. Significant differences in the RA of the genera are illustrated by ^*^ (Pairwise Wilcoxon rank-sum with Benjamini-Hochberg correction, *P* < 0.05).

Supplementary Figure 4 Bar plot showing the RAs of the genera of >1% RA of the total community from d 28 swabs by BRSV titer response for INJ and INT. Error bars display the standard error of the mean for each genera, and colors represent the BRSV response. The top nine most abundant genera are displayed in the legend. Significant differences in the RA of the genera are illustrated by ^*^ (Pairwise Wilcoxon rank-sum with Benjamini-Hochberg correction, *P* < 0.05). High responder = top 10 BRSV titer response from each trt group on d 28. Low responder = bottom 10 BRSV titer response from each trt group, usually 0 on d 28.

Supplementary Figure 5 Bar plot showing the RAs of the genera of >1% RA of the total community from samples on d 28, by health status. Error bars display the standard error of the mean for each genera, and colors represent the health status at the end of the feeding period; healthy, BRD cases, or mortalities. The nine most abundant genera are displayed. Significant differences in the RA of the genera are illustrated by different letters (Pairwise Wilcoxon rank-sum with Benjamini-Hochberg correction, *P* < 0.01).

Supplementary Figure 6 Bar plot showing the RAs of the genera of >1% RA of the total community from d 28 swabs by Clinical Illness Score (CIS) and fever status at the time of BRD diagnosis. The top bar plots show RA of microbial phyla, by CIS Score and fever status; the five most abundant phyla are shown in the legend. Bottom bar plots show the average RA of the six most abundant genera by CIS Score, with error bars representing the standard error of the mean. Significant differences in the RA of the genera are illustrated by ^*^ (Pairwise Wilcoxon rank-sum with Benjamini-Hochberg correction, *P* < 0.05).

Supplementary Table 1 PERMANOVA and PERMDISP results from comparisons of health status, day, and treatment based on generalized UniFrac distances. Significant results are bolded (*P* < 0.05). v., versus; CON, control; INT, intranasal; INJ, injected; Fin. Hlth. Stat., final health status; D, Day; Df, degrees of freedom; SS, sum of squares; p-adj., adjusted *p*-value.

Supplementary Table 2 Mean relative abundance, plus or minus the standard error of the mean, for taxonomic phyla representing >0.1% of the overall microbial community, vaccine treatment groups, and sampling day.

Supplementary Table 3 Mean relative abundance plus or minus the standard error of the mean of taxonomic phyla representing >0.1% of the overall microbial community across all samples at each of the sampling time points.

Supplementary Table 4 Mean relative abundance plus or minus the standard error of the mean of taxonomic genera representing >1.0% of the overall microbial community.

Supplementary Table 5 Mean relative abundance plus or minus the standard error of the mean of taxonomic phyla representing >0.1% and genera representing >1% of the overall microbial community across samples collected at the time of BRD treatment (*n* = 114).
