# Supplementary figures and images for: Effects of respiratory virus vaccination and bovine respiratory disease on the respiratory microbiome of feedlot cattle
Source: Front Microbiol. 2023 Jun 13;14:1203498. doi: 10.3389/fmicb.2023.1203498 (PMC10294429; doi:10.3389/fmicb.2023.1203498)

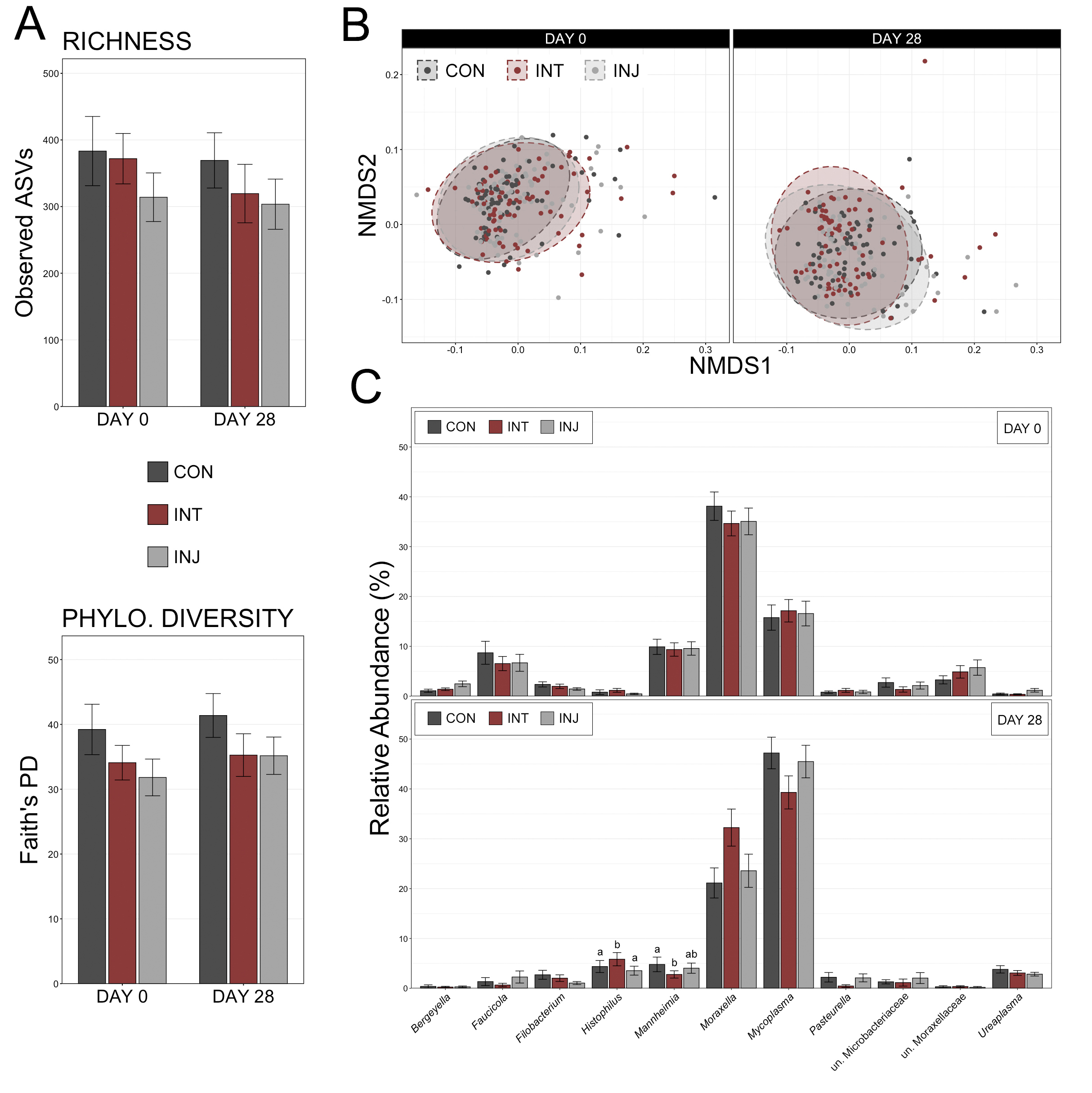

Supplement: Supplementary Figure 1 — (A) Boxplots displaying the number of observed ASVs and Faith's phylogenetic distance for samples from healthy cattle, by treatment and sampling day. Significant differences in richness and diversity between components are illustrated by different letters (Pairwise Wilcoxon rank-sum with Benjamini-Hochberg correction, P > 0.05). (B) Non-metric multidimensional scaling (NMDS) generalized UniFrac distances illustrating variation in microbial community structure associated with each treatment. The NMDS demonstrates clustering of 16S rRNA gene sequences by treatment in all animals. Dashed lines and shaded areas represent 95% confidence ellipses for each treatment. No significant difference in community structure was detected (pairwise PERMANOVA, P > 0.05). (C) Bar plot showing the RAs of the genera of >1% RA of the total community from all samples by treatment. Error bars display the standard error of the mean for each genera, and colors represent the treatment. The 10 most abundant genera are displayed in the legend. No significant differences in the RA of the genera were found. [file Image_1.JPEG]

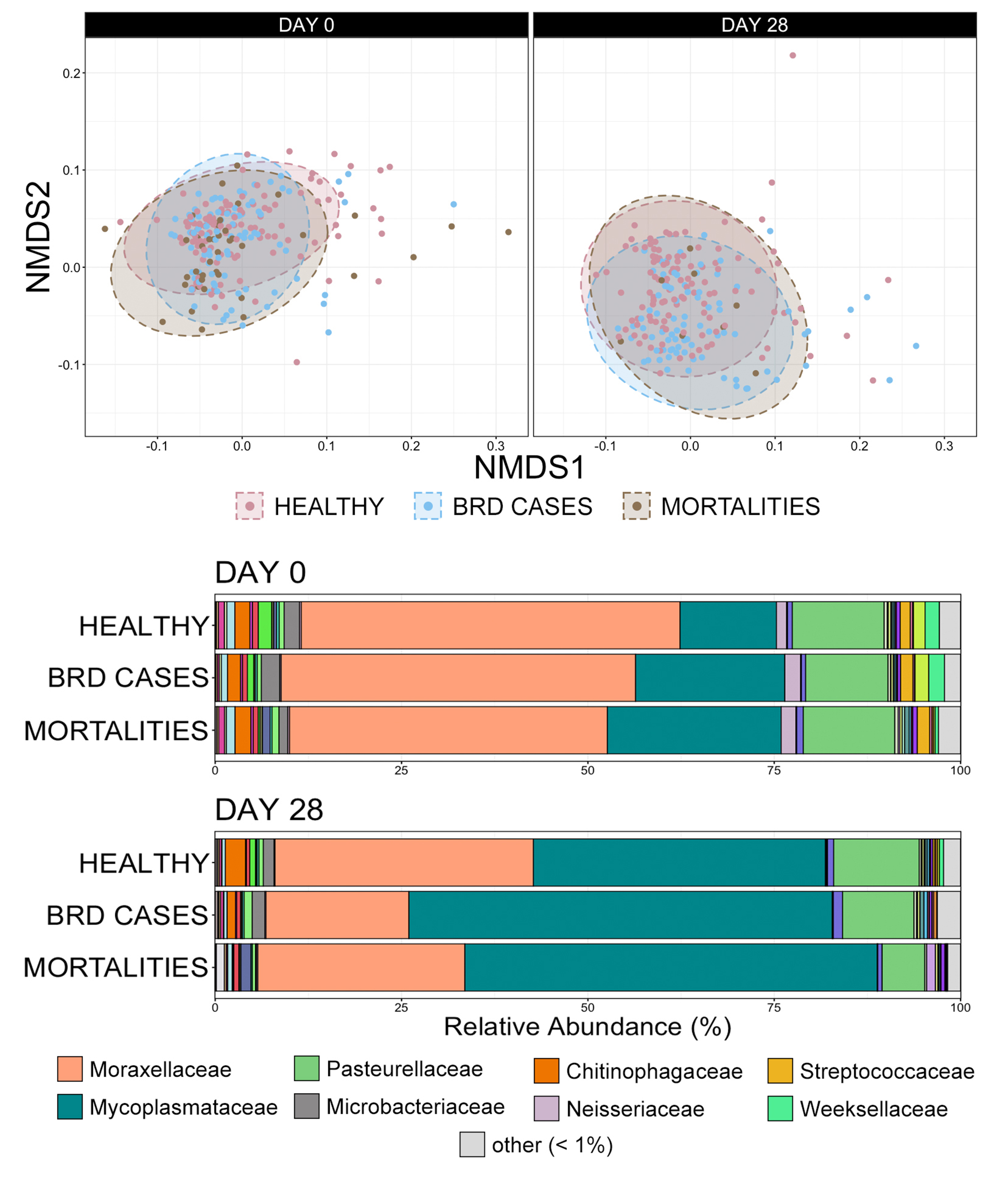

Supplement: Supplementary Figure 2 — Non-metric multidimensional scaling (NMDS) generalized UniFrac distances illustrating variation in microbial community structure associated d 0, stratified on final health classification. The NMDS demonstrates clustering of 16S rRNA gene sequences by health status among all animals. Dashed lines and shaded areas represent 95% confidence ellipses for each final health classification category. Bar plots showing the RAs of taxa found in the respiratory microbiota at the family level (RA > 1%) on d 0 and d 28, stratified on final health classification. Colors represent the different family taxa. The eight most abundant families are identified in the legend. [file Image_2.JPEG]

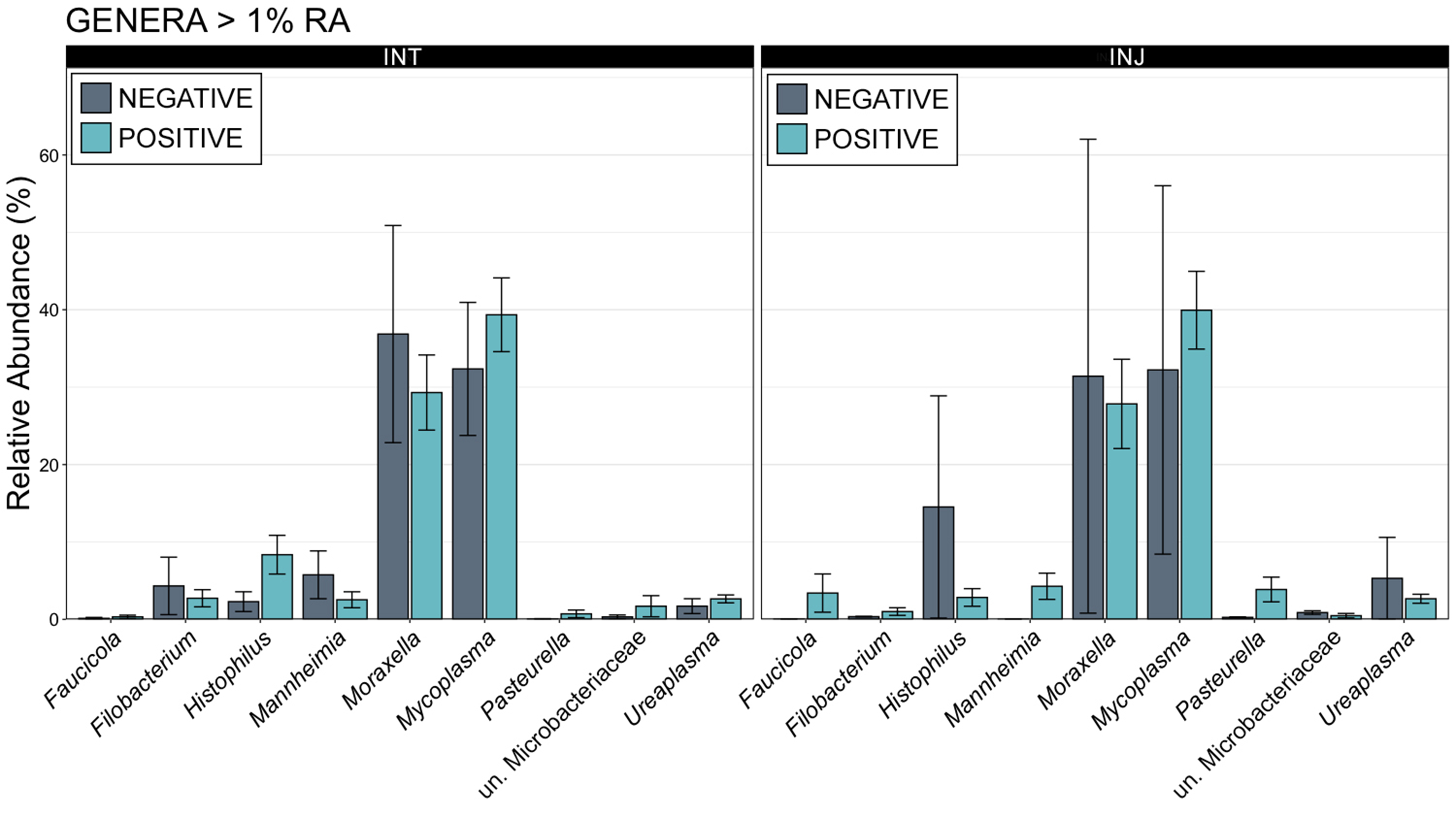

Supplement: Supplementary Figure 3 — Bar plot showing the average RAs of the genera with >1% RA of the total community from d 28 swabs, by vaccination group (INT vs. INJ) and serum BRSV titer status (seronegative vs. seropositive) on d 28. BRSV titer response for INJ and INT. Error bars display the standard error of the mean for each genera and colors represent the BRSV response. The nine most abundant genera are displayed in the legend. Significant differences in the RA of the genera are illustrated by * (Pairwise Wilcoxon rank-sum with Benjamini-Hochberg correction, P < 0.05). [file Image_3.JPEG]

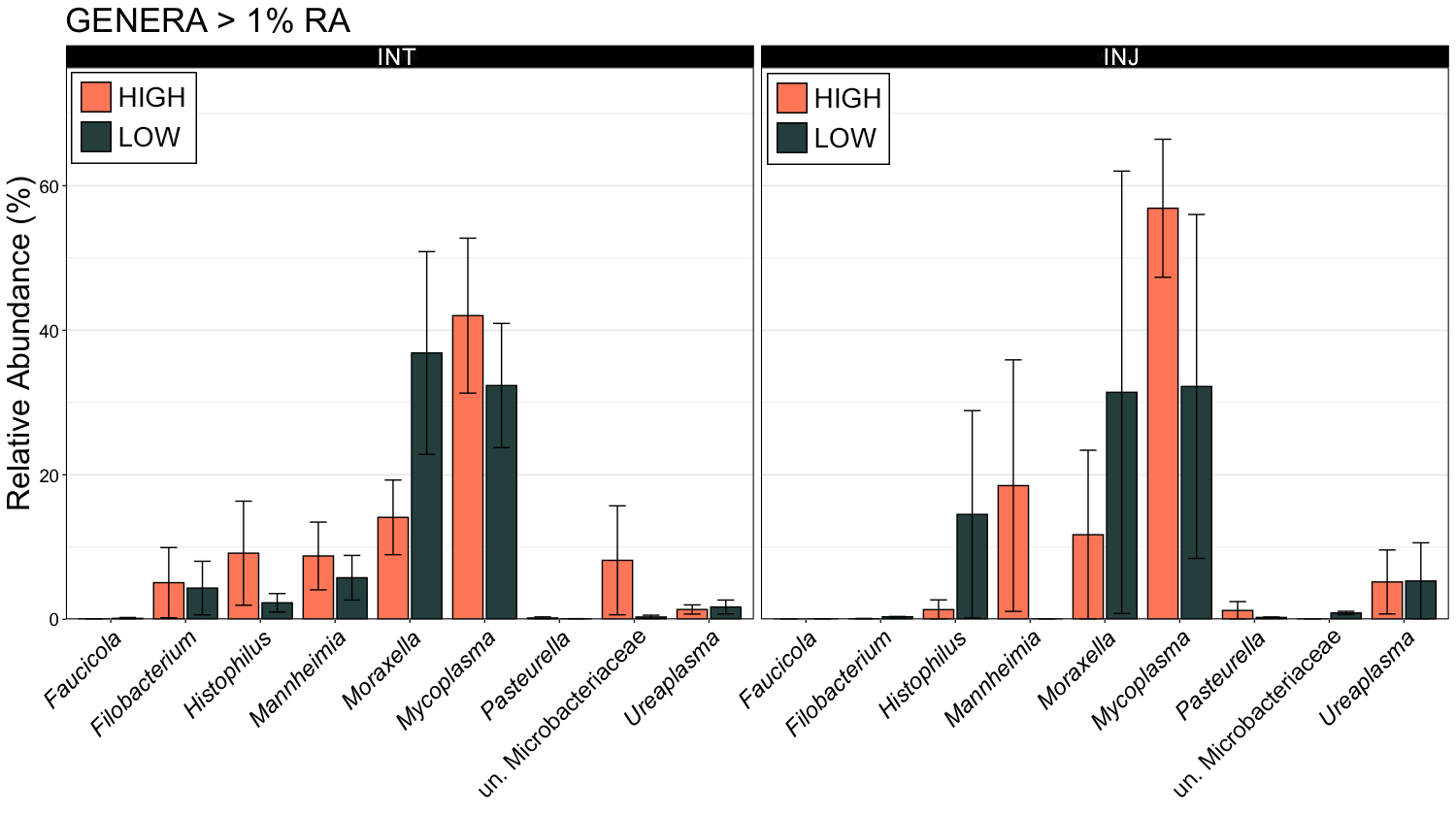

Supplement: Supplementary Figure 4 — Bar plot showing the RAs of the genera of >1% RA of the total community from d 28 swabs by BRSV titer response for INJ and INT. Error bars display the standard error of the mean for each genera, and colors represent the BRSV response. The top nine most abundant genera are displayed in the legend. Significant differences in the RA of the genera are illustrated by * (Pairwise Wilcoxon rank-sum with Benjamini-Hochberg correction, P < 0.05). High responder = top 10 BRSV titer response from each trt group on d 28. Low responder = bottom 10 BRSV titer response from each trt group, usually 0 on d 28. [file Image_4.jpeg]

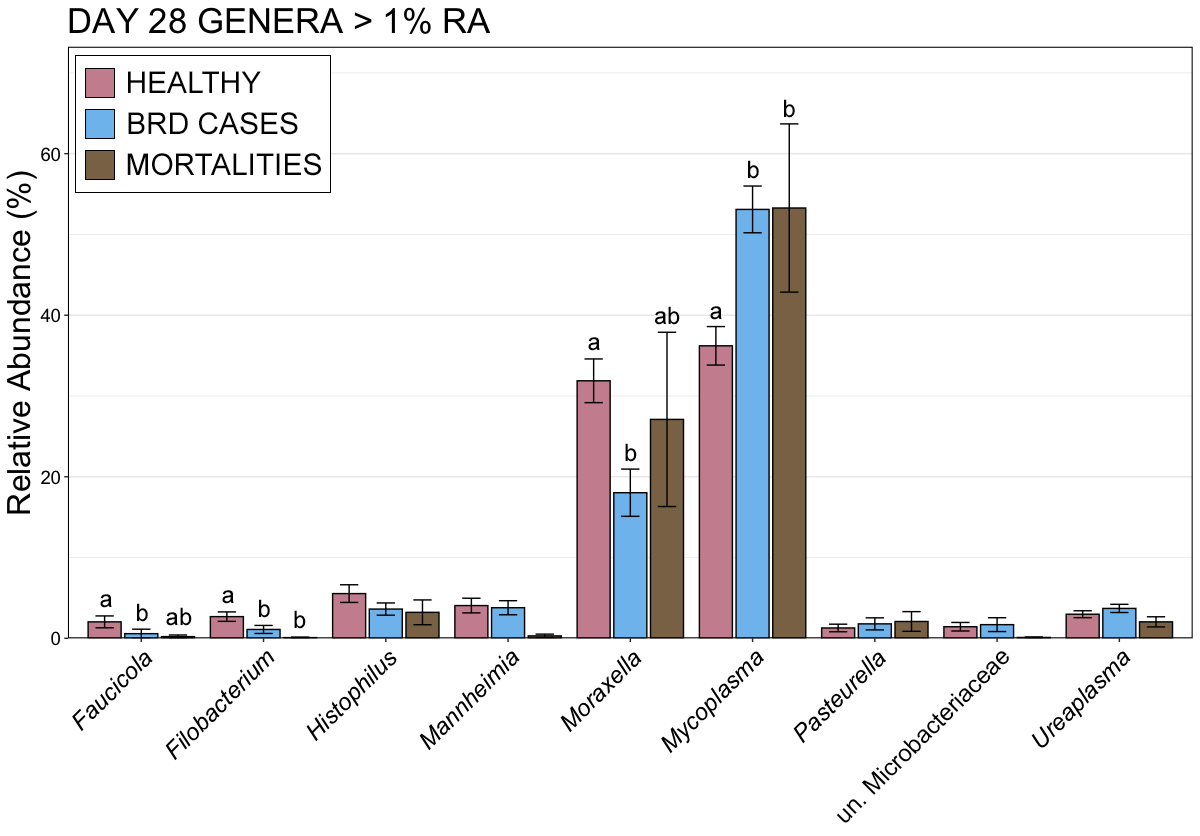

Supplement: Supplementary Figure 5 — Bar plot showing the RAs of the genera of >1% RA of the total community from samples on d 28, by health status. Error bars display the standard error of the mean for each genera, and colors represent the health status at the end of the feeding period; healthy, BRD cases, or mortalities. The nine most abundant genera are displayed. Significant differences in the RA of the genera are illustrated by different letters (Pairwise Wilcoxon rank-sum with Benjamini-Hochberg correction, P < 0.01). [file Image_5.jpeg]

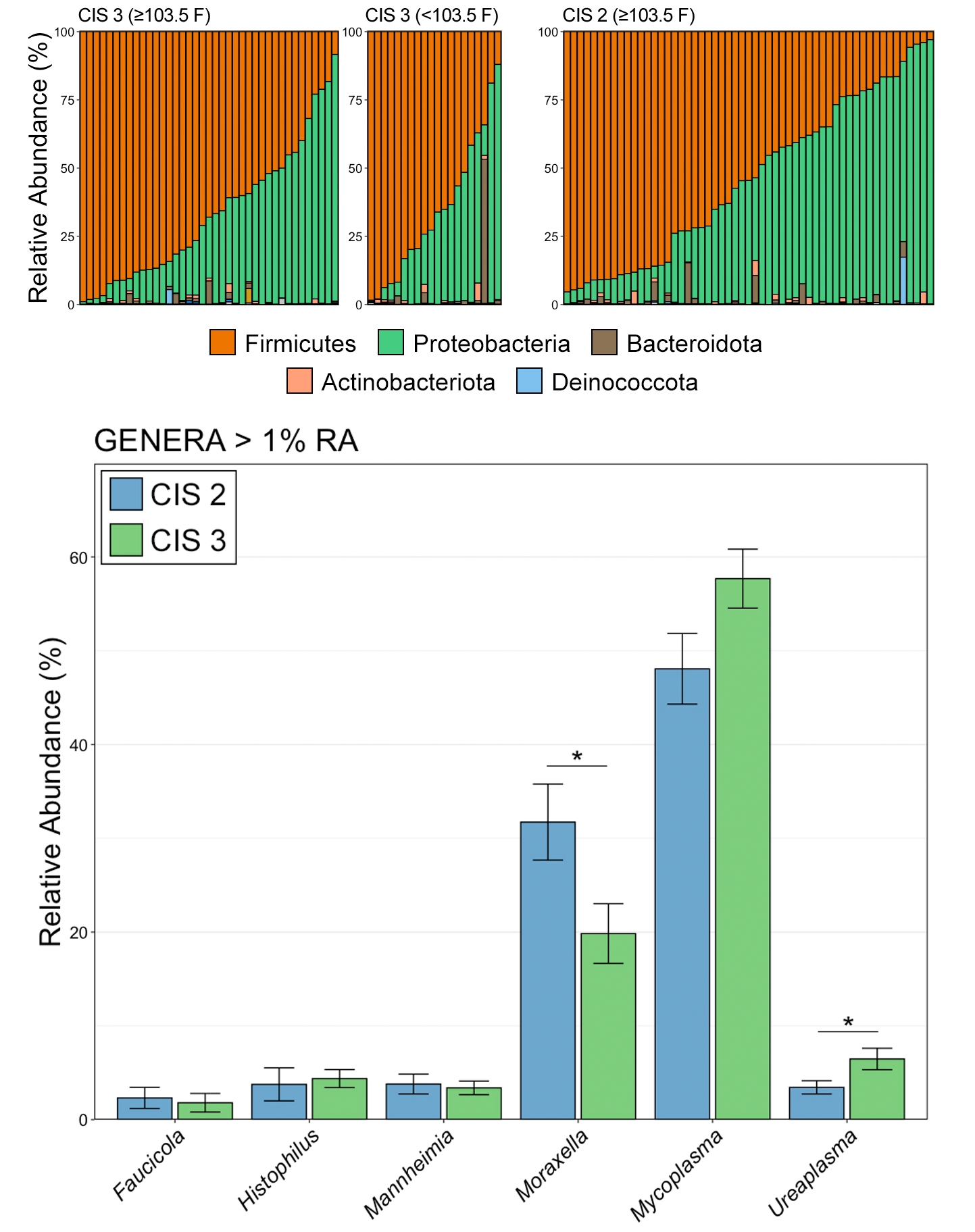

Supplement: Supplementary Figure 6 — Bar plot showing the RAs of the genera of >1% RA of the total community from d 28 swabs by Clinical Illness Score (CIS) and fever status at the time of BRD diagnosis. The top bar plots show RA of microbial phyla, by CIS Score and fever status; the five most abundant phyla are shown in the legend. Bottom bar plots show the average RA of the six most abundant genera by CIS Score, with error bars representing the standard error of the mean. Significant differences in the RA of the genera are illustrated by * (Pairwise Wilcoxon rank-sum with Benjamini-Hochberg correction, P < 0.05). [file Image_6.jpeg]
