# Supplementary material for: Effects of respiratory virus vaccination and bovine respiratory disease on the respiratory microbiome of feedlot cattle
Source: Front Microbiol. 2023 Jun 13;14:1203498. doi: 10.3389/fmicb.2023.1203498 (PMC10294429; doi:10.3389/fmicb.2023.1203498)
Supplement: Supplementary Table 1 — PERMANOVA and PERMDISP results from comparisons of health status, day, and treatment based on generalized UniFrac distances. Significant results are bolded (P < 0.05). v., versus; CON, control; INT, intranasal; INJ, injected; Fin. Hlth. Stat., final health status; D, Day; Df, degrees of freedom; SS, sum of squares; p-adj., adjusted p-value. [file Table_1.DOCX]

**Table S1**. PERMANOVA and PERMDISP results from comparisons of health status, day, and treatment based on generalized UniFrac distances. Significant results are bolded (P < 0.05).

| **Test** | **Df** | **SS** | **Pseudo-F** | **R^2^** | **p-adj.** | **PERMDISP (p-adj.)** |
| --- | --- | --- | --- | --- | --- | --- |
| **D0 v. D28 (Healthy)** | 1 | 0.6165 | 18.706 | 0.0761 | **0.001** | 0.448 |
| **D0 v. D28 (Morbid)** | 1 | 0.7660 | 23.898 | 0.1272 | **0.001** | 0.207 |
| **D0 v. D28 (Mortality)** | 1 | 0.1295 | 3.690 | 0.0714 | **0.002** | 0.505 |
| **HEALTHY D0** |  |  |  |  |  |  |
| CON v. INJ | 1 | 0.0373 | 1.211 | 0.0163 | 0.226 |  |
| CON v. INT | 1 | 0.0229 | 0.670 | 0.0091 | 0.813 |  |
| INT v. INJ | 1 | 0.0418 | 1.328 | 0.0163 | 0.165 |  |
| **HEALTHY D28** |  |  |  |  |  |  |
| CON v. INJ | 1 | 0.0358 | 1.086 | 0.0157 | 0.321 |  |
| CON v. INT | 1 | 0.0608 | 1.747 | 0.0225 | 0.060 |  |
| INT v. INJ | 1 | 0.0508 | 1.556 | 0.0201 | 0.090 |  |
| **MORBID D0** |  |  |  |  |  |  |
| CON v. INJ | 1 | 0.0310 | 1.070 | 0.0210 | 0.351 |  |
| CON v. INT | 1 | 0.0459 | 1.521 | 0.0269 | 0.102 |  |
| INT v. INJ | 1 | 0.0428 | 1.380 | 0.0229 | 0.145 |  |
| **MORBID D28** |  |  |  |  |  |  |
| CON v. INJ | 1 | 0.0394 | 1.117 | 0.0228 | 0.313 |  |
| CON v. INT | 1 | 0.0132 | 0.426 | 0.0081 | 0.969 |  |
| INT v. INJ | 1 | 0.0416 | 1.172 | 0.0205 | 0.263 |  |
| **FIN. HLTH. STAT. D0** |  |  |  |  |  |  |
| Healthy v. Morbid | 1 | 0.0541 | 1.723 | 0.0086 | 0.061 |  |
| Healthy v. Mortality | 1 | 0.0853 | 2.573 | 0.0162 | **0.009** | 0.635 |
| Morbid v. Mortality | 1 | 0.0549 | 1.705 | 0.0135 | 0.058 |  |
| **FIN. HLTH. STAT. D28** |  |  |  |  |  |  |
| Healthy v. Morbid | 1 | 0.1998 | 5.909 | 0.0299 | **0.001** | 0.986 |
| Healthy v. Mortality | 1 | 0.0609 | 1.814 | 0.0150 | **0.039** | 0.327 |
| Morbid v. Mortality | 1 | 0.0411 | 1.226 | 0.0139 | 0.255 |  |

Abbreviations: v., versus; CON, control; INT, intranasal; INJ, injected; Fin. Hlth. Stat., final health status; D, Day; Df, degrees of freedom; SS, sum of squares; p-adj., adjusted p-value
