# Supplementary material for: Effects of respiratory virus vaccination and bovine respiratory disease on the respiratory microbiome of feedlot cattle
Source: Front Microbiol. 2023 Jun 13;14:1203498. doi: 10.3389/fmicb.2023.1203498 (PMC10294429; doi:10.3389/fmicb.2023.1203498)
Supplement: Supplementary Table 2 — Mean relative abundance, plus or minus the standard error of the mean, for taxonomic phyla representing >0.1% of the overall microbial community, vaccine treatment groups, and sampling day. [file Table_2.DOCX]

| **CON D0**  **(n = 76)** | **INT D0**  **(n = 85)** | **INJ D0**  **(n = 82)** | **CON D28**  **(n = 62)** | **INT D28**  **(n = 77)** | **INJ D28**  **(n = 63)** | **Healthy CON**  **(D28; n = 35)** | **Healthy INT**  **(D28; n = 43)** | **Healthy INJ**  **(D28; n = 35)** |
| --- | --- | --- | --- | --- | --- | --- | --- | --- |
| Proteobacteria  65.01 ± 2.83 | Proteobacteria  61.09 ± 2.53 | Proteobacteria  61.72 ± 2.71 | Firmicutes  56.17 ± 3.17 | Firmicutes  47.99 ± 3.52 | Firmicutes  54.73 ± 3.23 | Firmicutes  49.34 ± 3.42 | Proteobacteria  53.69 ± 4.70 | Firmicutes  49.29 ± 4.55 |
| Firmicutes  24.15 ± 2.49 | Firmicutes  26.45 ± 2.26 | Firmicutes  26.39 ± 2.69 | Proteobacteria  35.40 ± 2.98 | Proteobacteria  43.67 ± 3.51 | Proteobacteria  37.22 ± 3.33 | Proteobacteria  40.41 ± 3.36 | Firmicutes  36.14 ± 4.29 | Proteobacteria  44.04 ± 4.59 |
| Bacteroidota  4.77 ± 0.63 | Bacteroidota  5.79 ± 0.84 | Bacteroidota  5.45 ± 0.70 | Bacteroidota  4.60 ± 0.95 | Bacteroidota  3.94 ± 0.74 | Bacteroidota  3.62 ± 0.58 | Bacteroidota  5.78 ± 1.37 | Bacteroidota  4.92 ± 1.17 | Bacteroidota  3.61 ± 0.69 |
| Actinobacteriota  4.65 ± 1.04 | Actinobacteriota  3.44 ± 0.60 | Actinobacteriota  4.23 ± 0.78 | Actinobacteriota  2.89 ± 0.48 | Actinobacteriota  2.80 ± 0.48 | Actinobacteriota  3.47 ± 1.13 | Actinobacteriota  3.10 ± 0.65 | Actinobacteriota  3.40 ± 1.36 | Actinobacteriota  2.07 ± 0.64 |
| Deinococcota  0.67 ± 0.21 | Deinococcota  2.02 ± 0.94 | Deinococcota  0.87 ± 0.49 | Deinococcota  0.43 ± 0.34 | Deinococcota  0.58 ± 0.28 | Deinococcota  0.32 ± 0.16 | Deinococcota  0.76 ± 0.60 | Deinococcota  1.02 ± 0.50 | Deinococcota  0.48 ± 0.28 |
| Patescibacteria  0.18 ± 0.06 | Fusobacteriota  0.57 ± 0.38 | Fusobacteriota  0.66 ± 0.64 | Verrucomicrobiota  0.18 ± 0.06 | Fusobacteriota  0.39 ± 0.22 | Verrucomicrobiota  0.12 ± 0.02 | Verrucomicrobiota  0.22 ± 0.11 | Chloroflexi  0.16 ± 0.12 | Fusobacteriota  0.10 ± 0.06 |
| Chloroflexi  0.11 ± 0.03 | Verrucomicrobiota  0.16 ± 0.03 | Patescibacteria  0.19 ± 0.06 |  | Chloroflexi  0.14 ± 0.07 |  |  | Fusobacteriota  0.15 ± 0.13 |  |
| Verrucomicrobiota  0.10 ± 0.03 | Patescibacteria  0.10 ± 0.03 | Verrucomicrobiota  0.10 ± 0.04 |  | Verrucomicrobiota  0.14 ± 0.04 |  |  | Verrucomicrobiota  0.14 ± 0.06 |  |
|  | Chloroflexi  0.10 ± 0.02 | Chloroflexi  0.10 ± 0.02 |  |  |  |  |  |  |

**Table S2.** Mean relative abundance plus or minus the standard error of the mean of taxonomic phyla representing > 0.1% of the overall microbial community within vaccine treatment groups.
