# Supplementary material for: Effects of respiratory virus vaccination and bovine respiratory disease on the respiratory microbiome of feedlot cattle
Source: Front Microbiol. 2023 Jun 13;14:1203498. doi: 10.3389/fmicb.2023.1203498 (PMC10294429; doi:10.3389/fmicb.2023.1203498)
Supplement: Supplementary Table 3 — Mean relative abundance plus or minus the standard error of the mean of taxonomic phyla representing >0.1% of the overall microbial community across all samples at each of the sampling time points. [file Table_3.DOCX]

| **All**  **(n = 559)** | **All D0**  **(n = 243)** | **All D28**  **(n = 202)** |
| --- | --- | --- |
| Proteobacteria  48.88 ± 1.22 | Proteobacteria  62.53 ± 1.55 | Firmicutes  52.60 ± 1.95 |
| Firmicutes  42.49 ± 1.25 | Firmicutes  25.71 ± 1.41 | Proteobacteria  39.12 ± 1.93 |
| Bacteroidota  4.09 ± 0.27 | Bacteroidota  5.35 ± 0.43 | Bacteroidota  4.05 ± 0.44 |
| Actinobacteriota  3.02 ± 0.27 | Actinobacteriota  4.11 ± 0.47 | Actinobacteriota  3.04 ± 0.48 |
| Deinococcota  0.74 ± 0.18 | Deinococcota  1.21 ± 0.38 | Deinococcota  0.46 ± 0.16 |
| Fusobacteriota  0.26 ± 0.12 | Fusobacteriota  0.43 ± 0.25 | Fusobacteriota  0.18 ± 0.09 |
| Verrucomicrobiota  0.12 ± 0.01 | Patescibacteria  0.16 ± 0.03 | Verrucomicrobiota  0.14 ± 0.02 |
|  | Verrucomicrobiota  0.13 ± 0.02 | Chloroflexi  0.10 ± 0.03 |
|  | Chloroflexi  0.10 ± 0.01 |  |

**Table S3.** Mean relative abundance plus or minus the standard error of the mean of taxonomic phyla representing > 0.1% of the overall microbial community across all samples at each of the sampling timepoints.
