# Supplementary material for: Effects of respiratory virus vaccination and bovine respiratory disease on the respiratory microbiome of feedlot cattle
Source: Front Microbiol. 2023 Jun 13;14:1203498. doi: 10.3389/fmicb.2023.1203498 (PMC10294429; doi:10.3389/fmicb.2023.1203498)
Supplement: Supplementary Table 4 — Mean relative abundance plus or minus the standard error of the mean of taxonomic genera representing >1.0% of the overall microbial community. [file Table_4.DOCX]

| **All**  **(n = 559)** | **All D0**  **(n = 243)** | **All D28**  **(n = 202)** |
| --- | --- | --- |
| *Mycoplasma*  33.78 ± 1.23 | *Moraxella*  35.88 ± 1.54 | *Mycoplasma*  43.66 ± 1.90 |
| *Moraxella*  30.26 ± 1.14 | *Mycoplasma*  16.52 ± 1.39 | *Moraxella*  26.14 ± 2.01 |
| *Mannheimia*  6.28 ± 1.45 | *Mannheimia*  9.61 ± 0.81 | *Histophilus*  4.68 ± 0.69 |
| *Faucicola*  4.08 ± 0.52 | *Faucicola*  7.27 ± 1.04 | *Mannheimia*  3.80 ± 0.62 |
| *Histophilus*  2.88 ± 0.34 | un. Moraxellaceae  4.67 ± 0.73 | *Ureaplasma*  3.23 ± 0.32 |
| *Ureaplasma*  2.48 ± 0.20 | un. Microbacteriaceae  2.05 ± 0.42 | *Filobacterium*  1.94 ± 0.39 |
| un. Moraxellaceae  2.29 ± 0.34 | *Filobacterium*  1.92 ± 0.24 | *Pasteurella*  1.52 ± 0.40 |
| *Filobacterium*  1.64 ± 0.18 | un. Neisseriaceae  1.68 ± 0.37 | un. Microbacteriaceae  1.48 ± 0.45 |
| un. Microbacteriaceae  1.47 ± 0.25 | *Bergeyella*  1.67 ± 0.24 | *Faucicola*  1.38 ± 0.47 |
| *Pasteurella*  1.05 ± 0.17 | *Streptococcus*  1.51 ± 0.19 |  |
|  | un. Lactobacillales  1.42 ± 0.22 |  |
|  | *Deinococcus*  1.21 ± 0.38 |  |

**Table S4.** Mean relative abundance plus or minus the standard error of the mean of taxonomic genera representing > 1.0% of the overall microbial community.
