# Supplementary material for: Effects of respiratory virus vaccination and bovine respiratory disease on the respiratory microbiome of feedlot cattle
Source: Front Microbiol. 2023 Jun 13;14:1203498. doi: 10.3389/fmicb.2023.1203498 (PMC10294429; doi:10.3389/fmicb.2023.1203498)
Supplement: Supplementary Table 5 — Mean relative abundance plus or minus the standard error of the mean of taxonomic phyla representing >0.1% and genera representing >1% of the overall microbial community across samples collected at the time of BRD treatment (n = 114). [file Table_5.DOCX]

| **Phylum** | **Genus** |
| --- | --- |
| Firmicutes  60.32 ± 2.62 | *Mycoplasma*  53.05 ± 2.47 |
| Proteobacteria  37.07 ± 2.62 | *Moraxella*  25.58 ± 2.61 |
| Bacteroidota  1.50 ± 0.50 | *Ureaplasma*  5.01 ± 0.69 |
| Actinobacteriota  0.65 ± 0.10 | *Histophilus*  4.08 ± 0.98 |
| Deinococcota  0.22 ± 0.16 | *Mannheimia*  3.59 ± 0.63 |
|  | *Faucicola*  2.05 ± 0.74 |

**Table S5.** Mean relative abundance plus or minus the standard error of the mean of taxonomic phyla representing > 0.1% and genera representing >1% of the overall microbial community across samples collected at the time of BRD treatment (n=114).
